# Long-term phenological trends, species accumulation rates, aphid traits and climate: five decades of change in migrating aphids

**DOI:** 10.1111/1365-2656.12282

**Published:** 2014-10-03

**Authors:** James R Bell, Lynda Alderson, Daniela Izera, Tracey Kruger, Sue Parker, Jon Pickup, Chris R Shortall, Mark S Taylor, Paul Verrier, Richard Harrington

**Affiliations:** 1 Department of AgroEcology, Rothamsted Research, West CommonHarpenden, AL5 2JQ, UK; 2 SASARoddinglaw Road, Edinburgh, EH12 9FJ, UK

**Keywords:** British aphid species checklist, gamm4, linear mixed-effects model, species discovery curves, suction-trap

## Abstract

**1.** Aphids represent a significant challenge to food production. The Rothamsted Insect Survey (RIS) runs a network of 12·2-m suction-traps throughout the year to collect migrating aphids. In 2014, the RIS celebrated its 50th anniversary. This paper marks that achievement with an extensive spatiotemporal analysis and the provision of the first British annotated checklist of aphids since 1964.

**2.** Our main aim was to elucidate mechanisms that advance aphid phenology under climate change and explain these using life-history traits. We then highlight emerging pests using accumulation patterns.

**3.** Linear and nonlinear mixed-effect models estimated the average rate of change per annum and effects of climate on annual counts, first and last flights and length of flight season since 1965. Two climate drivers were used: the accumulated day degrees above 16 °C (ADD16) indicated the potential for migration during the aphid season; the North Atlantic Oscillation (NAO) signalled the severity of the winter before migration took place.

**4.** All 55 species studied had earlier first flight trends at rate of β = −0·611 ± SE 0·015 days year^−1^. Of these species, 49% had earlier last flights, but the average species effect appeared relatively stationary (β = −0·010 ± SE 0·022 days year^−1^). Most species (85%) showed increasing duration of their flight season (β = 0·336 ± SE 0·026 days year^−1^), even though only 54% increased their log annual count (β = 0·002 ± SE <0·001 year^−1^).

**5.** The ADD16 and NAO were shown to drive patterns in aphid phenology in a spatiotemporal context. Early in the year when the first aphids were migrating, the effect of the winter NAO was highly significant. Further into the year, ADD16 was a strong predictor. Latitude had a near linear effect on first flights, whereas longitude produced a generally less-clear effect on all responses. Aphids that are anholocyclic (permanently parthenogenetic) or are monoecious (non-host-alternating) were advancing their phenology faster than those that were not.

**6.** Climate drives phenology and traits help explain how this takes place biologically. Phenology and trait ecology are critical to understanding the threat posed by emerging pests such as *Myzus persicae nicotianae* and *Aphis fabae cirsiiacanthoidis*, as revealed by the species accumulation analysis.

## Introduction

The Rothamsted Insect Survey (RIS) has been operating 12·2-m suction-traps since 1964 and celebrated its 50th anniversary on the 29th April 2014 with a series of research talks that examined the contribution of the RIS to ecological research (Harrington 2014). Its long-term data are of unquestionable importance, which increases with time, particularly given recent issues such as climate change, biodiversity loss and invasive species. In this paper, we use the RIS suction-trap network, the most comprehensive, standardized data set on terrestrial invertebrates in the world (Harrington *et al*. 2013), to address all those issues, showing how species have accumulated over the last five decades, how flight phenologies are changing for 55 aphid species and how such changes may be explained by their life-history traits and mechanisms related to a changing climate.

### The Rothamsted Insect Survey

#### Getting the right height

**Fig. 1:**
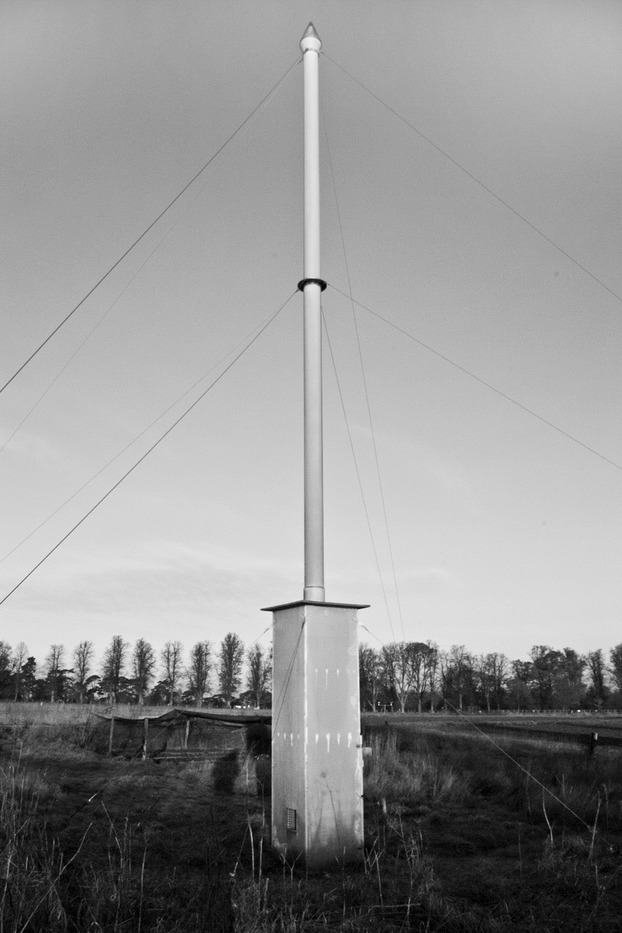
A suction-trap that samples migrating insects at a height of 12·2 m. The vertical 9·2-m pipe has an internal diameter of 244 mm. The 3-m housing at the base of the trap contains a motor and an electric fan from which a sample volume of 0·75 m^3^ air per second is generated. Aphids and other insects flying over the trap are pulled down through the vertical pipe into a collecting bottle inside the housing.

During development of the suction-trap network, Johnson (1957) and Taylor (1974a) showed, through an exhaustive set of height-density experiments, that insect density tends to decrease with height but that at greater heights aphids form proportionately more of the aerial insect fauna. From this, Taylor (1974a,b) derived mathematically the ‘logarithmic mean height of aphid flight’ which was shown to be 12·2 m. This height also yielded a suitable sized aphid sample that was representative of the general aerial population over a wide area. With this knowledge and some simple infrastructure (Fig.1), the 12·2-m traps have been sampling aphid migrations since the 1960s at a continuous rate of *c*. 45 m^3^ air per minute, without variation or bias at most commonly occurring wind speeds (Macaulay, Tatchell & Taylor 1988).

#### Responding to Silent Spring

The original remit of the RIS suction-trap network was to provide farmers with information on the timing and size of aphid migrations, particularly immigrations to cereals, where aphids were instigating heavy prophylactic use of insecticides, highlighted as an issue in 1962 by Rachel Carson in her book ‘*Silent Spring*’ (Woiwod & Harrington 1994; Harrington, Shortall & Woiwod 2010). By way of a response, the RIS began issuing weekly bulletins of the main aphid pests to the farming community as a means of reducing insecticide use through improved timing of application. This was a major contribution by the RIS to the issues surrounding *Silent Spring*. Currently, the RIS contributes data to pest forecasting, population dynamics and, more recently, biodiversity which all resonate with Carson's original concerns.

#### The RIS and its research themes over the last 50 years

As standardized data accumulated, a time series developed that became useful to researchers. Briefly, the use of RIS data has been to study neutral theory, spatial synchrony, genetic diversity and variability, the evolution of insecticide resistance, principles of migration and long-term trends under climate change. Pre-eminently, the discovery of Taylor's power law that describes mean–variance relationships and the application of it to RIS data is seen as a major contribution to ecology (Taylor & Woiwod 1982). For a comprehensive discussion of research associated with the RIS and its evolution see Woiwod & Harrington (1994), Harrington & Woiwod (2007), Harrington, Shortall & Woiwod (2010) and Harrington (2014).

In this paper, we focus on phenology (i.e. the study of seasonal timing of biological events) which has been a central theme for the RIS in the last 20 years (Clark *et al*. 1992; Fleming & Tatchell 1994; Worner, Tatchell & Woiwod 1995; Zhou *et al*. 1995; Cocu *et al*. 2005; Harrington *et al*. 2007; Harrington & Clark 2010). Phenology has been highlighted recently by Miller-Rushing *et al*. (2010), Diez *et al*. (2012) and Rafferty *et al*. (2013) as being critical to understanding changes in demography, species interactions and ecosystem processes. Many authors have linked shifting phenologies to changes in temperature, emphasizing the importance of this type of research for understanding the implications of climate change (Root *et al*. 2003; Musolin 2007; Altermatt 2012). However, climate may not have a consistent effect on species which may promote the decoupling of ecological interactions, termed ‘trophic mismatching’, that describes the developing asynchrony between species that depend on another for resources (Harrington, Woiwod & Sparks 1999; Visser & Both 2005; Musolin 2007; Thackeray *et al*. 2010). It is this mismatch that may ultimately weaken the resilience of ecosystems (Durant *et al*. 2007) although some species may not show immediate declines even with large mismatches (Reed *et al*. 2013). Aphids are only now being linked to trophic mismatching in their role as a food source for birds (S. Thackeray pers. comm.).

Aphids also represent a significant challenge to agriculture and are an economically important group because of the damage they cause to their plant hosts. In this regard, we also aim to elucidate aphid species accumulation rates in suction-trap samples in the context of the known UK aphid fauna, assessing how many species records remain outstanding, whether we should expect to find new potentially major pest species amongst this group and whether there are any implications for the suction-trap network. These strategic analyses are imperative for ecology because aphids are an excellent model group to study climate change adaptation. These analyses also contribute to our understanding of pest management.

## Materials and methods

### Suction-Traps

The suction-traps (Fig.1) continuously measure the aerial density of flying aphids, sampling at the logarithmic mean height of aphid flight (12·2 m) and provide daily records during the main aphid flying season (April–November) and weekly records at other times (Taylor 1974b; Macaulay, Tatchell & Taylor 1988).

### Species Accumulation

#### Traps and aphid species studied

**Fig. 2:**
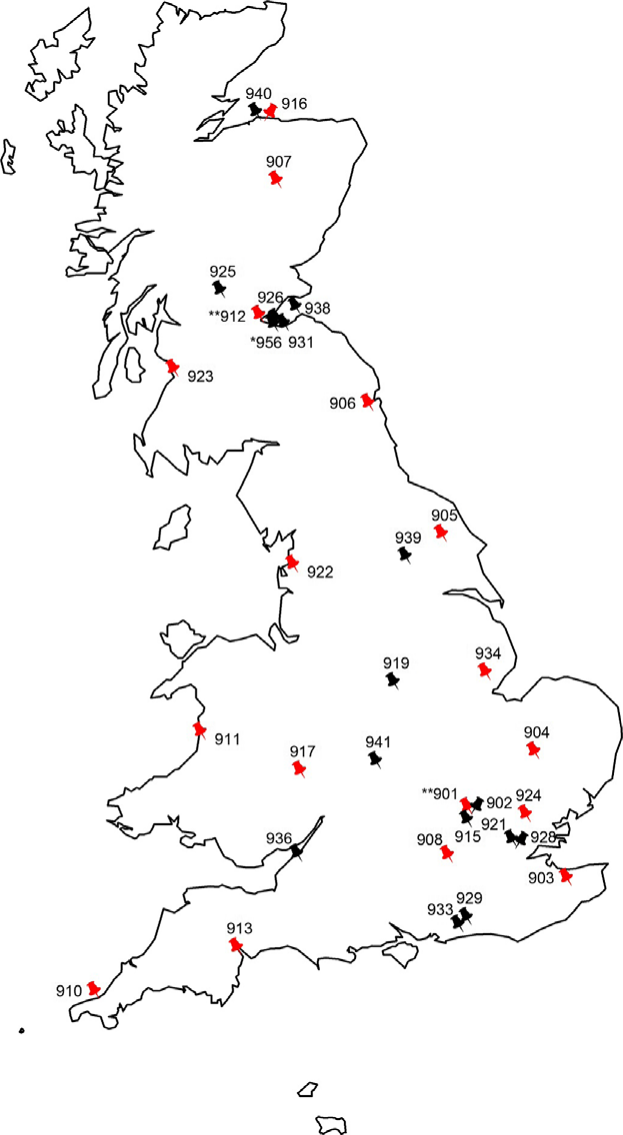
The location of the 37 UK 12·2 m suction-traps. The pin number refers to the Rothamsted Insect Survey (RIS) trap number that can be found in Appendix S1. As described in the methods, 37 traps were used for the species accumulation analysis that included all traps pictured. A trap number with * or ** indicate that there were one (*) or two (**) suction-trap(s) in close proximity in addition to the one that has been identified with a trap number (see Appendix S1). For the phenological rates of change models, a subset of traps highlighted in red were used which comprised: Aberystwyth 911, Ayr 923, Broom's Barn 904, Dundee 907, East Craigs 912, Elgin I 916, Hereford 917, High Mowthorpe 905, Kirton I 934, Newcastle 906, Preston 922, Rosewarne 910, Rothamsted Tower 901, Silwood Park 908, Starcross 913, Writtle 924 and Wye 903.

A total of 37 suction-traps (Fig.2) comprising 770 trap-years of data was used to estimate the rate at which ‘new’ species (i.e. first network record) were recorded. We present these data as annual rates of species accumulation over time from (i) the beginning of the RIS long-term data set in 1965, following an experimental ‘set-up’ period that started in 1964, and (ii) from 1970 by which time 215 species had been recorded representing approximately a third of the total species recorded in the UK (i.e. 34·1% of the 629 species). During the early period after 1966, when there were still only two traps running, new traps came on stream at a rate of between 1 and 5 traps per annum (total traps per year in the network were: 1967 = 7; 1968 = 8 and 1969 = 11). The 1970 start date is important because 15 suction-traps were running by this time, damping the effect of the Rothamsted and Broom's Barn traps that accumulated a large number of common species during the first couple of years. The study is not perfectly balanced in that the number of suction-traps varied over time. Importantly, transformation of species accumulation cannot be standardized to a catch per unit effort as new records are based on singletons (Bebber *et al*. 2007, 2012), and thus, we are cautious in our interpretation, merely seeking to identify suction-trap outliers that are recording more or fewer species than expected when compared with other traps of a similar length of service.

#### UK aphid species checklist

A revised and authoritative checklist of all aphids recorded in the UK (Appendix S2) is provided. RIS bulletin species have been highlighted, indicating that these species represent a major threat to UK agriculture/horticulture in most years. Also annotated on the checklist are three species classifications: those which have been recorded by the RIS (#) and included in the species accumulation curve; species that could, in time, be recorded by the RIS (*); and species which will probably remain ‘undetected’ by the RIS (∧) because either they do not produce alates (i.e. winged adults) and/or they produce alates but require host plant information to be certain of the species identification in the absence of DNA methods. This new, annotated checklist updates the list of 535 species provided previously by Kloet & Hincks (1964) and the later specialist publications by Stroyan (1966, 1972, 1979, 1984) and Blackman (2010). For taxonomic authorities of each species mentioned in this paper, we refer readers to Appendix S2.

#### Bayesian probability of a new species being recorded in the next year

We use Bayes' theorem to elucidate the conditional probability of recording a new species to the suction-trap network, given the uncertainties concerning the numbers of species to discover and the numbers of determinable species. The methodology is given in Appendix S3.

### Phenology and Abundance

#### Traps, aphid populations studied and meteorological stations used

Fifty-five aphids were studied using a network of 17 suction-traps (Fig.2 – those in red) located across England, Wales and Scotland (Appendix S1b). The start year of the time series began with Broom's Barn and Rothamsted suction-traps that provided historical data on aphids from 1965. The end year for the time series for all traps was 2010 as subsequently a few samples have not been identified, and thus, complete annual totals are not available for all sites. Using the RIS database, we derived the first and last flight (i.e. the first and last record of a species at a given trap in a given year) and the duration of flight season which equated to the number of days between the 5th and 95th percentile flight for each species at a given trap in a given year. We also derived total annual counts per species per trap per year. For all derived metrics, we imposed the requirement that the annual counts of aphids per species per trap-year were ≥20. Further, we only used species-trap-year data that were at least 20 years in length. This ensured that any of the metrics derived from these annual counts (i.e. first and last flight, duration of flight season) were robust. As an indication of the size of the analysis, the sum of all aphid counts was 11 664 001 and the total number of trap-years studied was 563 (Appendix S1b).

#### Biological and agricultural significance of population metrics used

There have been several published papers analysing first flight trends in suction-trap data, in part because this metric is a good proxy for measuring the effect of winter temperatures on the leading edge of a population (Worner, Tatchell & Woiwod 1995; Cocu *et al*. 2005; Harrington *et al*. 2007; Harrington & Clark 2010; Thackeray *et al*. 2010). Further, first flights may indicate a lack of outbreak potential if the first flight is late relative to crop development. The duration of aphid flight season (defined here as the number of days between the 5th and 95th percentiles) has rarely been studied, although both Fleming & Tatchell (1994) and Zhou *et al*. (1995) indicate some basic trends in what they term ‘flight period’ or ‘FLT’. Essentially, the duration of the flight season is expected to increase if the prevailing conditions continue to be conducive to population growth. In New Zealand, long flight seasons are associated with large peaks in flight activity (Stufkens *et al*. 2000). However, a long flight season is only relevant to agriculture if the host plant is susceptible to feeding damage – should the peak be late, then the impact of the immigrating aphid is diluted greatly. An early peak is particularly relevant to young plants that have a weaker resistance to pathogens and are thus more likely to be compromised by aphid-borne viruses (Katis *et al*. 2007). Aphids could conceivably do significant damage even in a small flight season window provided the host plant was susceptible (Dewar, Woiwod & Choppin de Janvry 1980). The last flight record confirms the finality of a migrating population and is used rarely. This is despite the potential link between last flights and changing temperatures in autumn and general aphid abundance at a time when plant viruses, such as BYDV, are often transmitted (Harrington & Clark 2010). In agricultural terms, the last flight signals the end of the autumn transmission of plant viruses to crops by aphid vectors. Importantly, both first and last aphid flights are functions of population size and may not necessarily be synchronized with the remainder of the leading or trailing edge of the population (Sparks, Roberts & Crick 2001; Bell *et al*. 2013). The total annual abundance of suction trapped invertebrates is of intrinsic interest to ecologists studying trophic ecology (Benton *et al*. 2002; Shortall *et al*. 2009) or impacts of climate change and spatial structuring (Westgarth-Smith *et al*. 2007). Annual counts of aphids could align with projected flight seasons, although aphids have an intrinsic rate of increase that could rapidly change the relationship between flight season length and annual counts (Dewar, Woiwod & Choppin de Janvry 1980; Kindlmann, Jarošík & Dixon 2007).

#### Biological traits used directly to model phenological responses

Patterns in aphid populations can be studied in terms of their traits (Mondor, Tremblay & Messing 2007; Bell *et al*. 2012). Here, we use three physiological, nominally scaled traits in a mixed model environment. These are (i) a trait describing host-alternation potential: monoecious (i.e. individuals are non-host-alternating) or heteroecious (i.e. individuals host-alternate between a primary winter host and an unrelated secondary summer host); (ii) a trait describing predominant mode of reproduction: holocyclic (i.e. sexual phase in the life cycle) or anholocyclic (i.e. no sexual phase) and lastly, (iii) a life cycle plasticity trait: that is either obligate (i.e. compulsory single reproductive type) or facultative (adaptive reproductive type – that is, more than one life cycle type possible). All trait information was originally derived from Heie (1980), Blackman & Eastop (1994, 2000, 2006).

#### Meteorological data

We paired meteorological stations with suction-traps (Appendix S1c) and derived accumulated degree days above 16 °C for each site-year or for first flight site-year-period (i.e. April-May) [Σ (daily average temperatures – 16 °C)]. The lowest known threshold that will allow the initiation of aphid flight is 11 °C, but clearly this is not appropriate for this study as it will incur false-positive flight days as many species' thresholds are much higher. Consequently, we adopt the upper level cited by Irwin, Kampmeier & Weisser (2007) of 16 °C for which we assume there is no temperature barrier for any of the 55 species studied here. For first flight, we use the accumulated day degrees above 16 °C for the combined months of April and May only. For remaining responses, we use the annual accumulated day degrees above 16 °C which approximates to the aphid season from April–October when temperatures above 16 °C commonly occur.

The North Atlantic Oscillation (NAO) is based on the difference of normalized sea level pressure between Lisbon (Portugal) and Reykjavik (Iceland). It is summarized by one index that records the strength and sign of the NAO, typically over the last 50 years between −5 and 5. We used the station-based winter index from December through March to represent the weather during winter for each site-year (Hurrell & National Center for Atmospheric Research Staff (2014)).

#### Statistical analysis of phenology

Three phenological responses that comprised first flight, last flight, duration of flight season along with log annual counts were studied. Linear mixed-effects (LME) models in the NLME library within the R statistical modelling language (R Core Team 2014) were used to estimate the fixed effects of

1. ‘Year’ to then quantify (i) the annual linear average rate of change across all aphids and (ii) individual species change coefficients using subsets of data for each species (i.e. species data frames in R). We refer to these models hereafter as ‘the rates of change’ models or the ‘RoC models’.
2. Traits. The full fixed model comprised three categorical variables [host-alternating potential (monoecious/heteroecious); predominant mode of reproduction (holocycly/anholocycly) and life cycle plasticity (obligate/facultative)]. These were used to explain patterns in phenology and log annual counts. We refer to this model hereafter as the ‘traits model’.

In summary, there were two LME models (RoC and traits models) for each of the four responses, the former derived annual rates of change and individual species coefficients and the latter attempted to attribute trait information to the patterns produced from phenological and log annual counts.

The random effects structure differed between models. Harrington *et al*. (2007) recognized that annual counts of aphids could be linked to measures of phenology. Larger migrations are likely to incur a higher probability of being caught, and thus, the analysis is susceptible to correlated errors. We model this artefact in the RoC model using a nested random effect structure: annual log counts were nested in trap, and a random intercept was specified. By doing so, each annual log count that was nested in trap was allowed to have a different mean value (specified in R as random = ∼1|trap/log annual count) – recognizing generally that some traps produce higher counts than others which increases the probability of a given species being caught. Using a call to anova within LME, we then compared this random effects structure with separate trap and log count terms as intercept-only models to show that the Akaike Information Criterion (AIC) for the nested structure was optimal (Zuur *et al*. 2009). Note that when annual counts were the response, annual counts were dropped as a nested term from the random effects.

The random effects for the traits model was specified as random = ∼1|trap/year/species name which recognized the need for different mean values for trap, year and aphid species, given that populations may vary across different traps and that there were also between year differences at the species level. Using year, as opposed to log annual counts, encapsulated both an element of log annual counts and variations in weather, etc., and was shown to be supported statistically using a call to anova within LME (see above). Thus, the fixed traits effects were generalized over all species-trap-years when testing responses. Each response was tested directly by starting with the full fixed and random effects model, then following a reverse model selection procedure removing the fixed trait term with the smallest non-significant (*P* > 0·05) *t* statistic (Zuur *et al*. 2009). The final solution in each model had at least one fixed effect. Model checking of the standardized residuals, fitted values and errors was performed routinely.

All models were fitted using restricted maximum likelihood (REML) that gives less biased variance estimates than maximum likelihood (Zuur *et al*. 2009). With fixed effects included in the model, REML precludes the use of both the AIC and log-likelihoods as measures of the goodness-of-fit (Welham & Thompson 1997). Consequently, in lieu of using AIC, we refer to estimated *r*^2^ where appropriate.

To aid interpretation of the species groupings, a neighbourhood-joining dendrogram was computed on individual species coefficients (Past version 1.99; Hammer, Harper & Ryan 2001). Jaccard similarities were used to group the responses together. To supplement these models and to reduce dimensionality of the individual species change coefficients from the RoC model, a biplot that included predictive axes from a principal components analysis (PCA) was used (Genstat 16th Edition; VSN International Ltd., Hemel Hempstead, UK) alongside a neighbourhood-joining dendrogram, both based on Euclidean distances. These two approaches represented the major features of the data, such as outliers that could not be captured with a Jaccard similarity.

#### Modelling aphid phenology along with climate drivers in a spatiotemporal context

To examine statistically the role of climate in driving the aphid responses (i.e. first flight, last flight, duration of flight season and log annual counts), we used the generalized additive mixed model package ‘gamm4’ that fits a Gaussian model to each response using REML (R Core Team 2014). There are three components to a gamm model: the fixed and random effects and the smooth terms (Wood 2006). The random effects structure was simply a generalization over species (random = ∼1|species). Spatial location (i.e. longitude; latitude) and year were included as covariates to be smoothed. By doing so, it suggested that these three covariates were of intrinsic interest in the study of climate rather than purely nuisance variables (cf site and year as random effects). The degree of smoothing of each of these terms was estimated by the model. The fixed effects structure was the accumulated degree days over 16 °C and the NAO. We were cautious in accepting any model, balancing the significance of the fixed effects and their *t* value, with the confidence intervals about the smoothed terms. We placed great emphasis on model performance using gam.check and vis.gam and supplementary box plots on the fixed effects. Throughout, *P* values on the 0·05 boundary were treated with some scepticism as to their true significance (Zuur *et al*. 2009).

## Results

### Species Accumulation Rate

**Fig. 3:**
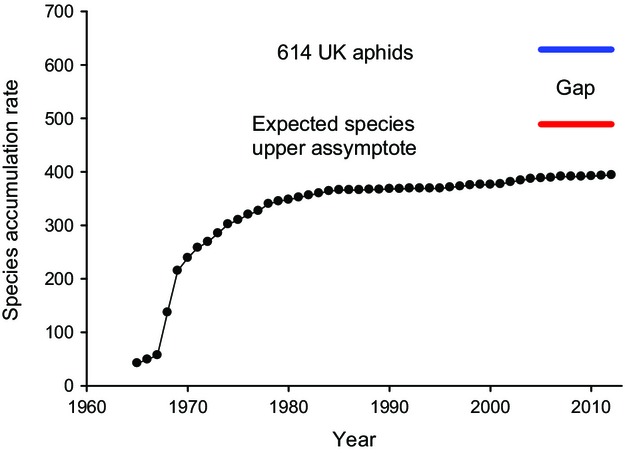
Species accumulation rate that shows the cumulative rate of new species recorded in the network per annum. The discovery gap between observed (dotted line) and the expected species discovery upper asymptote would require accumulating 89 more species to the 394 species recorded to date. Between the expected species discovery upper asymptote and the UK list that comprises 614 species (Appendix S2), there are 140 species that are unlikely to be sampled by the suction-traps either because they do not produce alates (winged adults) or they require host plant information to identify individuals.

The Bayesian probability of recording a ‘new’ species in the suction-trap network was about 80% for the next year (*P* = 0·793) (Appendix S3a). As an estimate, the average trap produces 0·529 new species per annum ± SE 0·172, but there are clearly biases (Appendix S3b). As expected, new species to the network are considerably more difficult to find as time passes (Fig.3). Whilst Rothamsted and Broom's Barn were the first traps to begin providing long-term data in 1965, Rothamsted has produced twice as many species. Silwood Park appears to produce many more species, particularly given that it has a fragmented time series that began in 1968, stopped in 1988 and began once more in 2000 at a time when new species to the network were considerably harder to find. Of the species that remain to be discovered, three subspecies are of economic importance: *Myzus persicae nicotianae*, *Aphis fabae cirsiiacanthoidis* and *Aphis fabae mordvilkoi* (Appendix S2).

### Phenology and Climate

#### Phenology of aphids and effect of climate over five decades

**Fig. 4:**
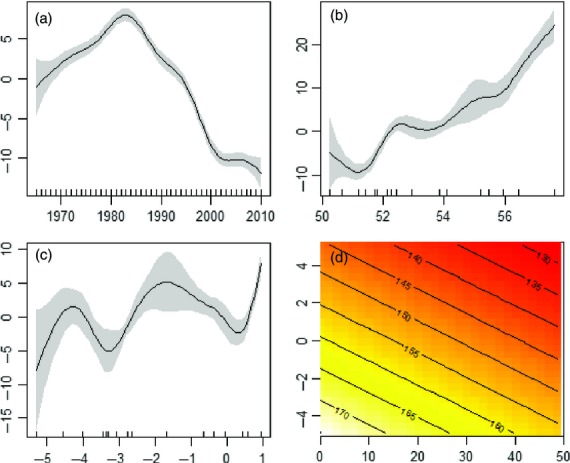
The first flight ‘gam’ component of the generalized additive mixed model that shows the smoothers related to year (a: effective degrees of freedom (edf) = 7·89), latitude (b: edf = 6·87), longitude (c: edf = 7·42) and the linear component relating the fixed effects of accumulated day degrees above 16 °C for the combined months of April and May and the North Atlantic Oscillation during winter (d). In plots a–c, the graphics show the estimated smoother effects with 95% confidence intervals. In these plots, the *y*-axis is the spatial smooth term according to the edf. The *x*-axis has two components; the major tick marks indicate numerical values and above those are rug plots that show the values of the covariates for each smooth. For year, the rug plots are regularly spaced but for latitude and longitude they are irregular.

Averaging out over all species, the LME RoC models showed that first flights were getting significantly earlier at a rate of −0·611 ± SE 0·015 days year^−1^ *r*^2^ = 0·465 (*t* = −40·716 *P* < 0·0001) but last flights appeared relatively stationary (−0·010 ± SE 0·022 days year^−1^ *r*^2^ = 0·558; *t* = −0·445 *P* = 0·656). The average flight season was getting significantly longer (0·3357 ± SE 0·02614 days year^−1^ *r*^2^ = 0·339; *t =* 12·842 *P* ≤ 0·0001) even though the log annual count was only moderately increasing (0·002 ± SE 0·0008 log annual count year^−1^ *r*^2^ = 0·539; *t* = 2·58404 *P* = 0·0098). As a spatial covariate in the additive climate models, year also acted on the aphid responses in a nonlinear way. All the LME estimations are correct in linear terms, but first flight analysis revealed a nonlinear component, being ‘humped’ around 1980 and thereafter showing a dramatic advancement (*F* = 165·2 *P* < 0·001; Fig.4a). Although stationary over the time period, last flights appeared to oscillate over time with roughly 20-year period length and with greater uncertainty during 1965–1970 (*F* = 15·30 *P* < 0·001; Appendix S4b). The average flight season did get longer over the whole time series, but there was a period between 1975 and 1995 when the response was relatively flat (*F* = 57·62 *P* < 0·001; Appendix S4b). Lastly, log annual counts showed a moderate increase, but a post-millennial trough suggests that these are dynamic (*F* = 9·24 *P* < 0·001; Appendix S4b).

**Fig. 5:**
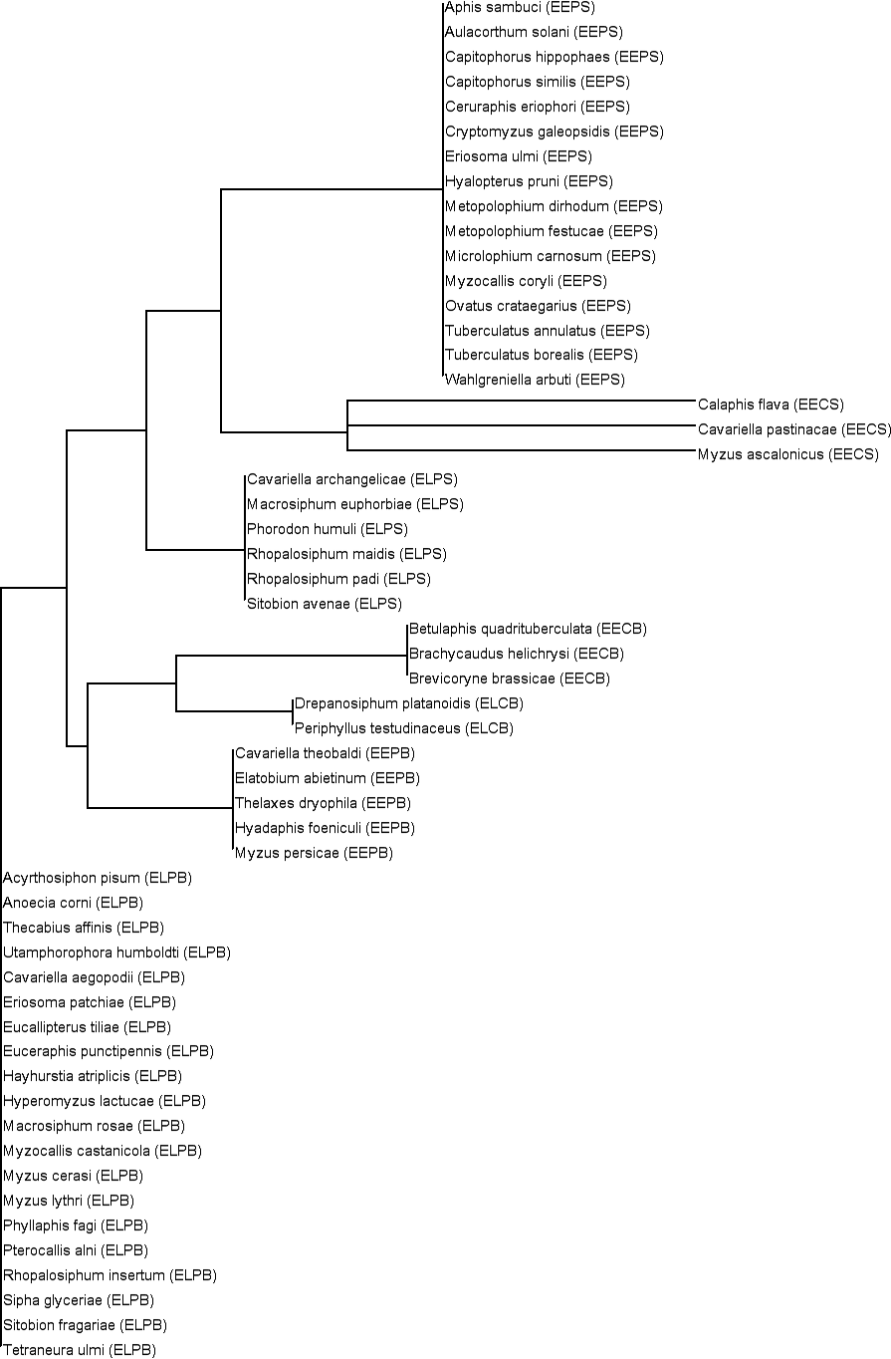
The species responses dendrogram based on a neighbourhood-joining algorithm with Jaccard similarities. The original matrix included 4 columns and 55 rows. The four responses (first flight, last flight, duration of flight season, log annual count) were columns, and the 55 rows were the species. The matrix was populated with the year coefficient from the linear mixed-effects models for each species. The neighbourhood-joining algorithm then produced the above dendrogram from the coefficients that displayed each species as a node. In parentheses along with the species, name is a categorical description of the trends for each response annotated as a four letter code [e.g. *Phorodon humuli* (ELPS)]. Each character is related to a response in the following order with the character underlined that indicates the direction in the trend: first flight [Earlier or Later]; last flight [Earlier or Later]; duration of flight season [Contracted or Protracted]; log annual count [Smaller or Bigger]. Thus, *P. humuli* (ELPS) has an earlier first flight, later last flight, a protracted flight season and a smaller log annual count trend. A complementary analysis using Euclidean distances to detect outliers is presented in Appendix S5.

Both the accumulated degree days above 16 °C (ADD16) during April and May and winter climate were linked to the advancement of aphid first flights (Fig.4). Per unit change in degree days yielded an advance of 0·379 days ± SE 0·025 (*t* = −14·74; *P* < 0·001), but the previous winter had a much stronger effect leading to 2·93 days ± SE 0·089 earlier (*t* = −32·89; *P* < 0·001) per unit change of the NAO. The combined effects of these climate variables can be seen in Fig.4d: warm wet winters, signalled by positive NAO values, combined with very high ADD16 values led to very early first flights in spring (i.e. area of deep red interpolation). As the aphid flight season accumulated degree days above 16 °C, the duration of the flight season contracted (0·078 days ± SE 0·005; *t* = −13·59; *P* < 0·001) and the effect of cold winter appeared to extend the duration of the flight season (2·261 days ± SE 0·163; *t* = 13·86; *P* < 0·001). The effect of winter was not significant by the time that the last flight is realized (*t* = −1·441; *P* > 0·05). The temperature effect through the year accumulated but brought forward, rather than pushed back, the last flight (0·043 days ± SE 0·004; *t* = −8·814; *P* < 0·001), similar to the effect on the duration of the flight season that measures the 5th–95th percentiles. The effect of climate on log annual counts produces an unconvincing gamm model perhaps because the response itself oscillates considerably between years and across species (Appendix S4b). Looking in more detail at the intercept of species random effect component (intercept = 0·991; standard deviation = 0·996), it is clear that there is very high variation in the species responses when the fixed effects are zero. The fixed effect of the NAO is close to the 0·05 significance boundary (*t* = −2·546; *P* < 0·05) and should be treated with caution (Zuur *et al*. 2009). Second, whilst ADD16 produces a highly significant result, the per unit change in ADD16 produces very small increments to the log count (0·001 log aphids ± SE <0·001; *t* = 6·921; *P* < 0·001). We cautiously accept that increasing the accumulated degree days will produce small, positive changes in annual counts recognizing that this model also struggles to explain the wide species variation (Fig. 5).

The spatiotemporal covariates in all the above gamm models suggest that aphid phenology was significantly determined by where and when the event takes place. Latitudinal effects were clear and close to linear for first flight (Fig.4b *F* = 101·80 *P* < 0·001). As first flights progressed northwards, they took place later in the year. The further south, the longer the duration of the flight season was (*F* = 43·90 *P* < 0·001) although aphid populations become distinctly nonlinear in terms of last flight (*F* = 26·57 *P* < 0·001) and log annual count (*F* = 26·57 *P* < 0·001, Appendix S4b). In both cases a noisier, nonlinear response occurred northwards from 54°, approximately where the Preston trap is located (Fig.2; Appendix S4b). First and last flights also appeared to have an opposing pattern in terms of longitude: in the west, first flights were earlier, but last flights were earlier in the east (first: *F* = 17·7 *P* < 0·001; last: *F* = 10·19 *P* < 0·001 – Fig.4c). Longitudinal effects were close to the boundary for log annual counts and appear to have wide confidence intervals (*F* = 3·03 *P* < 0·01) and although this spatial influence is stronger for the duration of flight season (*F* = 9·31 *P* < 0·01), longitude had a nonlinear effect.

#### Species responses

The log annual count data for many species suggest that they oscillate around a baseline linear trend that indicates either no long-term change is apparent or a very marginal upward trend is detectable, but both with substantial year-to-year variation (Appendix S4a). This is particularly true of pest species such as *Brachycaudus helichrysi*, *Brevicoryne brassicae*, *Metopolophium dirhodum* and *Sitobion avenae* whose annual counts are determined by many within-year processes that are not necessarily carried over to the following year. There is a small minority, such as *Anoecia corni* and *Tetraneura ulmi*, that are less volatile in terms of the long-term trend in log annual counts, but this is not a common response amongst the group (Appendix S4a).

*Utamphorophora humboldti* showed the most dramatic advancement in first flights (−2·709 days year^−1^) and also the most dramatic shift to later in the year of all last flights (2·713 days year^−1^). Consequently it also had the largest increase in the duration of flight season (2·531 days year^−1^) and largest population increase. Its outlier status is captured in the PCA and neighbourhood-joining dendrogram (Appendix S5). Two other species highlighted as outliers in the dendrogram are *Myzus ascalonicus* and *Periphyllus testudinaceus. Myzus ascalonicus* is advancing its first flight very slowly (−0·087 days year^−1^), has an earlier last flight (−1·900 days year^−1^) and, like *P. testudinaceus* (−0·901 days year^−1^), is contracting its flight season length (−0·896 days year^−1^). Curiously, even though *P. testudinaceus* has a shorter flight season, it has later last flights (0·529 days year^−1^), suggesting that there is a longer period at the end of the year when a small number of aphids remain in flight.

Considering all the phenological responses, there were seven groups of aphids defined by neighbour joining (Fig.5). Overall, there was not one group in which all are pests (see Appendix S2), or all have a common host or trait profile, or are equally rare or common. In short, the groups are well mixed although the highest density of pests is grouped as ‘ELPS’ (i.e. those that have earlier first flights, later last flights, protracted flight seasons and smaller log annual counts) bar *Cavariella archangelicae* in the ELPS group that feeds on umbellifers and does not have pest status. By far the largest group, with 20 species, is the ‘ELPB’ group [i.e. those that have earlier first flights, later last flights, protracted flight seasons and bigger annual counts (Fig.5)]. Six of these species are pests (*Acyrthosiphon pisum, Cavariella aegopodii, Hyperomyzus lactucae, Myzus cerasi, Rhopalosiphum insertum (Rhopalosiphum oxycanthae), Sitobion fragariae*). The common trait profile amongst 10 of the 20 species is for obligate heteroecious holocycly. The second largest group (EEPS) comprises 16 species that are either obligate heteroecious and holocyclic (six species) or obligate monoecious and holocyclic (six species). Even though their last flights are getting earlier and populations smaller, three pests can be found in this group; *Aulacorthum solani* and *Hyalopterus pruni* are pests of potatoes and plums, respectively, and *M. dirhodum* can cause damage to wheat. Of the remaining four smaller groups, the EECB group with three species that include two major pests, *B. helichrysi* and *B. brassicae*, and the EEPB group that comprises five species, two of which are major pests (*Elatobium abietinum*, *M. persicae*) are notable because both groups show earlier last flights.

### Traits

The trait ecology was found to explain some of the variability in observed patterns in first and last flight, duration of flight season and the log annual count of aphids (Table 1). A formal single traits mixed model analysis showed that generalizing across all species, heteroecious species tend to have a significantly longer duration to their flight season, with later last flights and larger log annual counts, although their first flights also tend to be later than for monoecious species (Table 1). Anholocycly promotes significantly earlier first and last flights, shorter flight seasons and smaller log annual counts than holocycly. A lack of life cycle plasticity incurring a compulsory single reproductive type (i.e. obligate) produces a first flight that is significantly later and a last flight significantly earlier with no significant increase in the duration of the flight season. The log annual count for a compulsory single reproductive type aphid was also significantly smaller.

**Table 1:** The fixed effects of traits in explaining the variation observed in the four responses given a nominal random effects structure (∼1|trap/year/species name)

	LME fixed effects		
LME response	Host plant alternation. Reference level: Holocyclic	Reproductive strategy. Reference level: Monoecious	Life cycle plasticity. Reference level: Obligate
1. First flight	2·316 ± SE 0·560***	−8·274 ± SE 0·437***	15·182 ± SE 0·614***
2. Last flight	6·400 ± SE 1·034***	−24·028 ± SE 0·809***	−5·262 ± SE 1·135***
3. Duration of flight season	5·171 ± SE 0·983***	−16·319 ± SE 0·768***	2·085 ± SE 1·079
4. Log annual count	0·551 ± SE 0·055***	−0·243 ± SE 0·028***	−1·026 ± SE 0·039***

The table includes the marginal significance (*P* < 0·001***) derived from conditional *t* tests for each of the three fixed coefficients. For each fixed effect, the trait of interest has a reference level which is compared against the remaining ‘free level’. In each case, these were (i) a host-alternating potential trait: heteroecious, the ‘free level’, was compared with a monoecious reference level; (ii) predominant mode of reproduction trait: anholocyclic, the ‘free level’, was compared with a holocyclic reference level; (iii) life cycle plasticity trait: facultative, the ‘free level’, was compared with an obligate reference level. If coefficient values were negative, the reference level in question is lower than the ‘free level’ and vice versa. Thus, holocyclic aphids have significantly later first flights (2·316 on the log_10_ scale) than anholocyclic aphids but monoecious aphids have significantly earlier first flights (i.e. −8·274 on the log_10_ scale) compared with heteroecious aphids. In all cases, bar life cycle plasticity in explaining duration of flight season, traits were highly significant in explaining observed responses in aphid migration characteristics.

## Discussion

### Phenology and Climate Change

The awareness that aphid population dynamics were influenced by climate was first expressed by Charles Elton (1927, p. 141), ‘Now suppose one of these small herbivores – a mouse or an aphid – is suddenly able to accelerate its rate of increase, either as a population or as an individual. The change might be caused by a favorable winter which would enable the population to start in spring with a larger capital of numbers than usual'. Elton had great foresight because the link between aphids and temperature, particularly winter temperature, is statistically strong and has the effect of changing their rate of population growth and flight phenology, amongst other population characteristics (Clark *et al*. 1992; Fleming & Tatchell 1994; Worner, Tatchell & Woiwod 1995; Zhou *et al*. 1995; Dixon 1998; Cocu *et al*. 2005; Harrington *et al*. 2007; Harrington & Clark 2010). Dixon (1998) argued that the projected increases in global temperatures were likely to have a positive impact on the developmental and reproductive rates but overall may not produce higher abundances *per se*. This conjecture was investigated by Newman (2005) who modelled populations of *Rhopalosiphum padi* under predicted CO_2_, temperature and rainfall patterns using HadRM03 projections. He predicted that this species would decline under current climate change projections, although as shown in this paper, long-term trends in abundance are complex and noisy, and no account was made in Newman's research for clonal evolution and adaptation. The lack of an overall population increase might be caused in some locations by hot summer temperatures that promote more winged aphids (alates) that do not contribute to overall metapopulation growth as dramatically as apterae (wingless aphids) (Michaud 2010). Further, temperature may cause mortalities to increase above a given threshold (≈30 °C) which is an additional constraint on growth (Dixon 1998; Irwin, Kampmeier & Weisser 2007).

Despite significantly advancing phenologies and longer flying seasons, aphid abundances generally are not increasing dramatically with year. Instead, numbers fluctuate widely between years indicating that that there are strong within-season processes that regulate the overall population size. Benton *et al*. (2002) explained the variation they observed in insect abundances from suction-traps as being highly correlated with agricultural practice, particularly intensity. We did not include these landscape variables but instead found that winter and summer climate drivers are either weak or not significant, indicating that there is much more to do when it comes to modelling aphid abundances, particularly with deterministic models (Kindlmann, Jarošík & Dixon 2007). The lack of a trend in increasing aphid abundance is consistent with what has been found for other groups that have been recorded from suction-traps. Shortall *et al*. (2009), for example, showed that total aerial biomass either decreased or showed no trend, depending on site. Overall, we found that abundances oscillate around a trend that suggests that they are, on average, marginally increasing although as Newman (2005) predicted, *R. padi* is not one of those aphids for which populations are growing.

Large-scale phenological changes have been observed and linked to climate change in marine, freshwater and terrestrial environments from sea level to the tops of mountains (Menzel *et al*. 2006; Thackeray *et al*. 2010; Burrows *et al*. 2011; Chapman 2013). Aphids are able to adapt to climate change faster than many other insect groups studied because of their low developmental threshold temperature and high intrinsic rate of increase (Harrington *et al*. 2007; Thackeray *et al*. 2010). We observed dramatic changes in the flight phenology of aphids, and we showed that these trends can be explained by the effect of climate, particularly that related to winter conditions as measured by the NAO. The NAO influences the mean wind speed, heat and moisture and has measureable effects on the overall European climate over both short and long time periods, particularly during the winter months when the atmosphere in this region is most active (Stenseth *et al*. 2003). Generally, animals and plants have been shown to respond to these annual NAO fluctuations, changing their reproductive phenology, spatial distribution, diversity and abundance as well as modifying interspecific relationships within communities that include predator–prey dynamics and competition (Coulson *et al*. 2001; Benton *et al*. 2002; Stenseth *et al*. 2003; Fisher *et al*. 2008). Green spruce aphid (*E. abietinum*) population dynamics have also been linked to the NAO: Westgarth-Smith *et al*. (2007) found that in a positive NAO phase, the spring migration will start earlier and the duration of the migration period will increase and contain many more individuals. However, the mechanism by which the NAO operates through the system was more complex than just winds conducive to migration. Strong emphasis was placed on the role of temperature anomalies associated with the NAO, later confirmed by nonlinear modelling (Lima, Harrington & Saldana 2008). Further, given that the NAO had a climate forcing effect on populations, Saldaña, Lima & Estay (2007) were able to link the NAO to levels of spatial synchrony. However, Westgarth-Smith *et al*. (2007) were unable to place any emphasis on more detailed precipitation changes associated with the NAO, even though these have been shown to affect the growth of pine, its host, within NAO phases. Further work is required, but the effect of the NAO on aphids is felt particularly at the extremes of the index, positive or negative. The effect of the NAO is reduced in years when the index is around zero, or negatively or positively weak. This has the tendency to show no discernible pattern in aphids. Hence, the winter NAO index will remain important in aphid population dynamics only if large values in the index (i.e. very cold, dry winters or very wet warm winters) continue to occur.

Temperature has a strong deterministic effect on whether aphids will migrate. The lowest threshold that will allow the initiation of flight is between 11 and 16 °C depending on species, which may be quickly exceeded in spring and very occasionally even in January in the UK (Irwin, Kampmeier & Weisser 2007; Met Office 2011). We found that as the degree days above 16 °C accumulate aphids respond by advancing both their first and last flight as well as contracting the duration of their flight season. Our results show that 16 °C is of ecological significance to the vast majority of aphid migrations. However, for both temperature and the accumulation of degree days above 16 °C, a nonlinear spatiotemporal effect is evident. Specifically, in most cases, there was strong latitudinal and temporal covariation. In our models, this translates as climate explaining the rates of change in phenology, when a strong north-south gradient and temporal sequence has been taken into account. Harrington *et al*. (2007) observed this in aphid first flight phenologies showing that longitude had a weaker effect on 16 aphid species across Europe but that latitude was a strong modifier. These observations remain true for the phenological metric studied here; in addition, we would add that spatial covariation in models of aphid abundance seems less likely to provide an insight into those populations' dynamics than do the within-year drivers (e.g. natural enemies and disease as regulators, host plant quality, etc.,). To that end, our abundance models suggest that more species-specific models, perhaps with a stochastic element to account for some unknowns, are required.

### Aphid Traits and Climate Change

In the face of climate change, what determines the rate at which aphids will respond to a warmer world? In this study, we showed the value of aphid traits in predicting outcomes. The most adaptive species should be those that are reproductively plastic, species which have continual parthenogenesis but also adaptive reproductive types that could, if required, go into a sexual phase. As facultative anholocyclics, *U. humboldti* and *Macrosiphum rosae* fit with this prediction, showing that they are benefitting from earlier first flights, later last flights, longer flight seasons with larger populations in time (Fig.5). Our traits analysis showed that anholocycly promotes significantly earlier first and last flights, shorter flight seasons but smaller annual counts than holocycly. Reproductive plasticity yields first flights that are significantly earlier and last flights significantly later, with larger populations in time although there was no significant increase in the duration of the flight season. We do not have enough ecological information on androcycly, a term first used by Blackman (1971) to describe a clone in which males and viviparous (i.e. parthenogenetic) females are produced. In both androcycly and anholocycly, the benefit of adaptation to a warmer world under climate change is via the parthenogenetic trait that maintains overwintering in the motile phase rather than eggs. Androcycly allows this trait to be passed through the sexual phase via males.

Dixon (1998) indicated that the evolution of anholocycly has a clear reproductive advantage because it doubles the intrinsic rate of increase relative to those with a sexual phase although he makes the point that its long-term evolutionary status seems less certain. Nevertheless, bet hedging through the inclusion of both sexual and parthenogenetic clones within a species (i.e. facultative anholocyclics) is most likely to be the optimum strategy for UK aphids that experience volatile winter temperatures (e.g. UK winters 2012–13 compared with long-term temperature averages, Met Office 2013), but not necessarily elsewhere (Halkett *et al*. 2004). Mondor, Tremblay & Messing (2007) suggested that successful aphid colonists of the Hawaiian Islands were anholocyclic. Indeed, European species that had adaptive reproductive types were shown by Harrington *et al*. (2007) to capture high variances in first flight models (see Harrington *et al*. 2007- Table 5. AH mon herb; AH het). In winter, motile aphids can quickly respond to changing climate compared with the egg stage produced from a sexual phase in the holocyclic aphid life cycle (Harrington *et al*. 2007; Harrington, Shortall & Woiwod 2010). Other facultative anholocyclic aphids included in our analysis (*Macrosiphum euphorbiae*, *M. persicae* and *Wahlgreniella arbuti*) all benefit to some degree from their suite of traits by showing protracted flight seasons, although there is variation in their responses in terms of whether their last flights and annual abundances are responding positively with time.

Host-alternation as a trait may increase fitness directly or indirectly by helping avoidance of parasitoids by changing the seasonal host. Only 10% of all aphids express this trait (Dixon & Kundu 1994; von Dohlen & Moran 2000; Williams & Dixon 2007). We showed that heteroecious (i.e. host-alternating) aphids tend to have a significantly longer flight season, with later last flights and larger annual counts although first flights also tend to be later than for monoecious species. Both nutrition and development can explain the differences in phenology for host-alternating species: firstly, these aphids are maximizing the availability of nitrogen-rich phloem in their primary hosts (i.e. trees/shrubs) in spring which is likely to lead to a delayed first flight migration, and secondly, host-alternators tend also to have very high fecundities (which affect annual counts) to offset the losses from migrating between hosts (Dixon & Kundu 1994; Ward *et al*. 1998; Bell *et al*. 2012). Higher annual abundances may help explain some of the phenological changes observed in this study because last flights and, to a degree, duration of flight season are functions of overall population size (Harrington & Clark 2010; Bell *et al*. 2013). However, constraining these aphids ecologically is the area (occupancy) of their winter host, indicating that they are very much tied into their primary host environment (Bell *et al*. 2012).

### Species Accumulation Rate

The suction-trap network has continuously measured the aerial density of flying aphids over the last five decades. In this period, the network has accumulated 81·5% of the expected species but recognizes that there is a discovery gap of 140 species that are unlikely to be sampled by (or identified from) the suction-traps either because they do not produce alates (winged adults) or they require host plant information to identify individuals (Appendix S2). Of the latter, DNA methods may contribute to identifying those aphids that otherwise require host plant data for accurate identification, although before it becomes routine, technical difficulties (e.g. quantitative molecular methods that yield individual counts that align with historical identification methods) need to be addressed. This particularly concerns subspecies of economic importance that we cannot verify without these molecular tools. For example, *Myzus persicae nicotianae* and *Aphis fabae cirsiiacanthoidis* may be important vectors of plant viruses to UK crops. We are in no doubt that given their prevalence elsewhere, they remain cryptically concealed within the archive, but will emerge as new records to the RIS within a very short period of time.

Suction-trap species data showed a classical accumulation rate: an early exponential phase in which a large number of new species was added to the network by a relatively small number of suction-traps was then followed by a levelling out of species accumulation rates post-1980 when a large number of traps were in operation (Bebber *et al*. 2007, 2012). For 2014 and beyond, we showed that the Bayesian probability of recording a ‘new’ species in the suction-trap network is about 80% in the next year, the average trap producing 0·529 new species per annum ± SE 0·172. New species are likely to come from exotic or rare host plants (e.g. *Takecallis taiwana* – on bamboo; *Tinocallis ulmiparvifoliae* and *Tinocallis zelkowae* – imported ornamental bonsai elms), glasshouses (e.g. *Cerataphis lataniae* – on fan palms and raffia palms; *Cerataphis orchidearum* – glasshouse orchids) and perhaps even unusual habitats such as coastal grasslands (e.g. *Trama maritima; Dysaphis maritima*) and wetlands (e.g. *Cavariella aquatica*; *Muscaphis cuspidata*). However, many of these species are likely to be difficult to catch if the host or habitats remain rare.

As we demonstrated, location of suction-traps is key if expected accumulation rates are to be maintained. Not all traps are equal, and some are predisposed to catching disproportionate numbers of species because of their location. For example, whilst Rothamsted and Broom's Barn were the first traps to provide data in 1965, Rothamsted has produced twice as many species. Silwood Park generally outperforms all other traps, perhaps because it is the only existing trap in parkland and located near a conurbation in which there is a strong ‘garden effect’. In practical terms, the species accumulation demonstrates the effectiveness of the suction-trap network whilst indicating that in the future, location is key for sampling invasive, exotic or rare species. Southerly towns and cities are most likely to yield new invasive, exotic or rare species records.

### Constraints of the Analysis

Whilst the RIS suction-traps provide one of the most robust sampling techniques available, there are aspects for which variation in life stages introduces a level of uncertainty that cannot easily be parameterized. One of the limits of this study is that we did not capture the within-season migration dynamics that can produce several migration phases, particularly for host-alternators. Taylor *et al*. (1981) showed that there are regional factors that can produce one, two or more migration phases within a species (e.g. *Phorodon humuli; R. padi*). Inherent within aphids is the fact that multiple migration phases overlap and become difficult to classify. A further complication is highlighted by Nottingham, Hardie & Tatchell (1991) who showed that autumn migrants had a longer migratory phase and a greater initial rate of climb compared with spring and summer migrants. There is potential for this variation in flight performance to affect the probability of capture in our suction-traps. In short, it must be recognized that annual counts, as used in this study, cannot capture any changing patterns of migration that may occur within a year (e.g. large spring migrations coupled with small autumn migrations vs. small spring migrations coupled with large autumn migrations). Both these flight patterns could yield the same annual count although they might have profoundly different implications for agriculture.

## Data archive

The Rothamsted Insect Survey and SASA hold long-term entomological data that represent a considerable investment in scientific endeavour. Data are held centrally on a database named ‘Paul’. Data requests can be made through a RIS/SASA member of staff, but these data are complex and require a high level of expertise to interpret. Whilst we are committed to sharing these data for *bona fide* scientific research, this will usually be through collaboration. For large or complex data or sample requests, Rothamsted Research/SASA reserves the right to recover the full marginal economic cost from the collaborator. Archive samples are available from 1974.

## Supporting Information

**Appendix S1.** Species, sites and years matrix and meteorological stations used (vsn 10).

**Appendix S2.** An annotated checklist of aphids present in the UK.

**Appendix S3.** Bayesian aphid probabilities and species accumulation.

**Appendix S4.** Individual aphid species time-series panel plots and gamm models.

**Appendix S5.** Principal components analysis of linear mixed-effects model coefficients.
