# Supplementary material for: Long-term phenological trends, species accumulation rates, aphid traits and climate: five decades of change in migrating aphids
Source: J Anim Ecol. 2014 Oct 3;84(1):21–34. doi: 10.1111/1365-2656.12282 (PMC4303923; doi:10.1111/1365-2656.12282)
Supplement: Supplementary file 3 — Appendix S3. Bayesian aphid probabilities and species accumulation. [file jane0084-0021-sd3.docx]

**Appendix 3a: The Bayesian Probability Of Incurring A New Species in the *nth* Year**

***P(H)***

The probability that *H* is correct in the absence of any other information, where *H* is the unconditional species discovery rate of determinable species (i.e. the rate at which new aphids are recorded in the UK per annum using suction trap data). We consider that the appropriate 'rate' is that which follows after the period of major discovery when new species have settled down to a stable rate. Thus, rather than starting the period at 1965, the start year is 1980 when 'new species' fluctuate between 0-5 records (see figure below). In the period 1980-2012, there are 10 years when no new species occurred but there are also 23 spearate years when at least 1 new species was added to the checklist (Appendix 2). Therefore, ***P(H)*** 23/33 expresses the unconditional probablity of recording a new species (i.e. ***P(H)*** =0.696) in 2014 and the *nth* if the status quo remains.

***P(E|H)***

The probability that *E* would be found if *H* were correct where *E|H* is the conditional probability of finding a 'determinable' new species from the whole of the British list. There are 629 species of aphid in the UK of which the RIS determines 394 known species. However, not all species found are determined to the species level because the genera *Aphis* (61 spp) *Uroleucon* (18 spp), *Dysaphis* (13spp), *Pemphigus* (9 spp) and *Cinara* (25spp) are not separated by the RIS and there are also some other undetected species too (see Appendix 2 - species annotated with a circumflex ^). Thus 394/629 suggest that the probability that *E|H* is correct is 0.626

***P(H_unknown_)***

This is simply (1-***P(H)*** ) or the probability of the unknown species discovery rate ***P(H)*** and is therefore equal to 1-0.696 (i.e 0.304)

***P(E|H_inaccurate_)***

The probability that *E* would be found it *H* were inaccurate. In other words, how precise is the RIS in identifying an individual to species level? In short, the RIS identifies species with an accuracy of 394/629 *P*=0.626) and thus is potentially underestimating new species by 1-0.626 (i.e. 0.374)

Bayes theorem is:

$$P\left( H | E \right)=\frac{P\left( H \right)\times P(E|H)}{\left( P\left( H \right)\times P\left( E | H \right) \right)+ (P\left( H_{unknown} \right)\times P\left( E | H_{inaccurate} \right))}$$

Substituting the values we have

$$P\left( H | E \right)=\frac{0.696 \times0.626}{\left( 0.696 \times0.626 \right)+(0.304 \times0.374)}$$

$$P\left( H | E \right)= \frac{0.435}{\left( 0.435 \right)+(0.113)}$$

$$P\left( H | E \right)=\frac{0.435}{0.548}$$

Thus the probability of finding a new species in 2014 is:

$$P\left( H | E \right)=0.793$$

This probably is an inflated probabilty of ***P(H)***. It is arguably more accurate because it estimates the effect of the known unknown (i.e. the species that we know we cannot identify).

**Appendix 3b : Species accumulation**

Figure The relationship between the length of time a suction-trap has been running and its species accumulation rate. Filled circles use data from the start of the RIS in 1965, filled triangles remove the first 5 years of data and start in 1970 by which time 215 species representing a third of the total species recorded (34.1%) had been discovered. Filled circles and triangles overlap for all traps less than 20 years in length apart from Farm 1 which ran for five years ceasing service in 1973 and recorded all but one of its 33 species during 1965-70. The year in which a trap joined the network is clearly important because common species inflate the rates of those early suction-traps. Notice how Farm I, Rothamsted and Silwood drop in accumulation rates when the time series starts in 1970 (i.e. note shift ● →▲). Even so, both Rothamsted and Broom's Barn were recording in 1965 but show differences, with Broom's Barn recording half the species caught at Rothamsted. Silwood was switched on 3 years after the start of the network, When the time series was curtailed to 1970, Silwood shows a bias for capturing more new records than any other trap. Given these differences, spatial location is clearly important. Generally many of the southern traps absorb a larger slice of the new species.
