# Supplementary material for: Long-term phenological trends, species accumulation rates, aphid traits and climate: five decades of change in migrating aphids
Source: J Anim Ecol. 2014 Oct 3;84(1):21–34. doi: 10.1111/1365-2656.12282 (PMC4303923; doi:10.1111/1365-2656.12282)
Supplement: Supplementary file 4 — Appendix S4. Individual aphid species time-series panel plots and gamm models. [file jane0084-0021-sd4.pptx]

## Slide 1
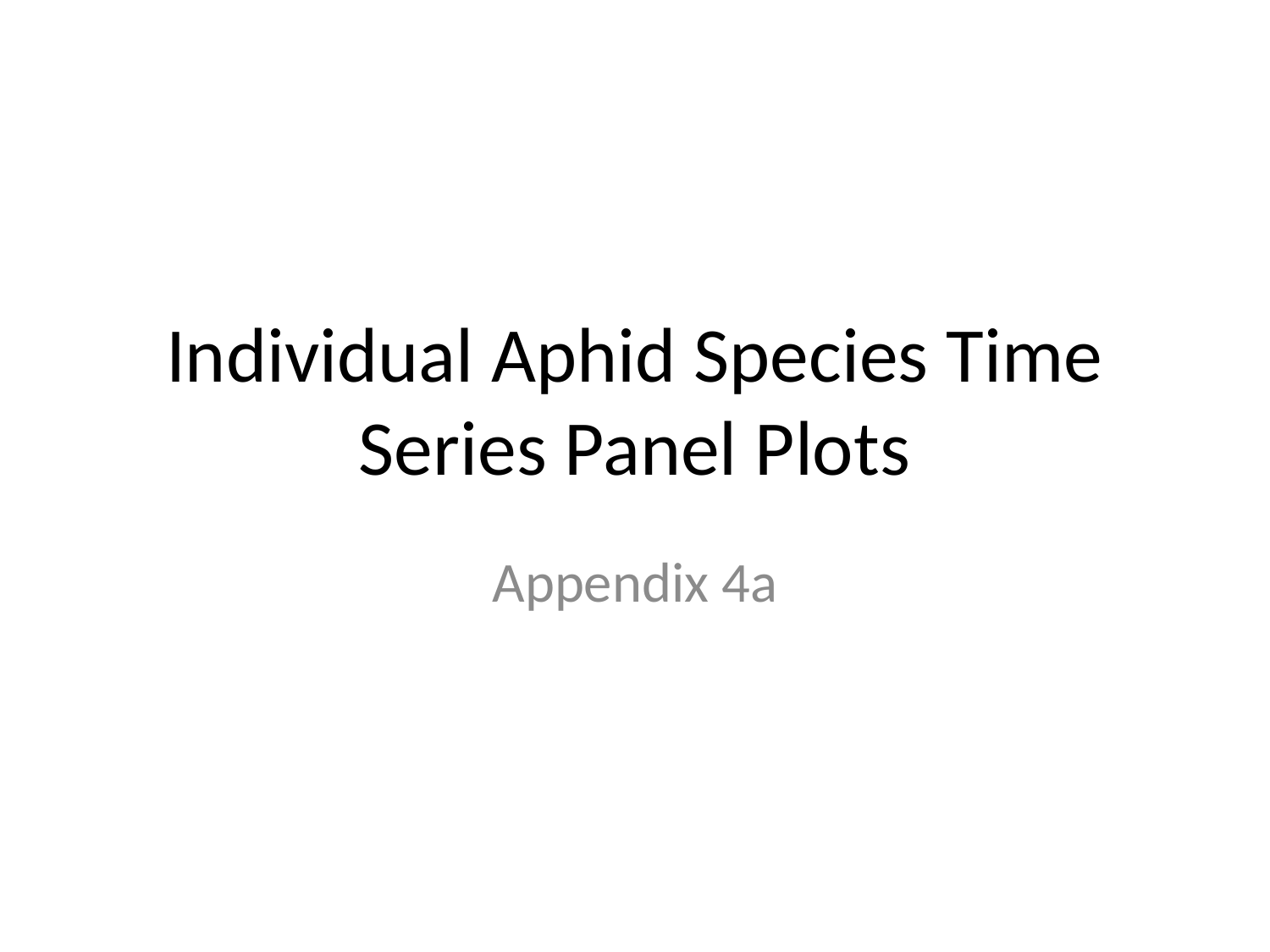

# Individual Aphid Species Time Series Panel Plots
Appendix 4a

## Slide 2
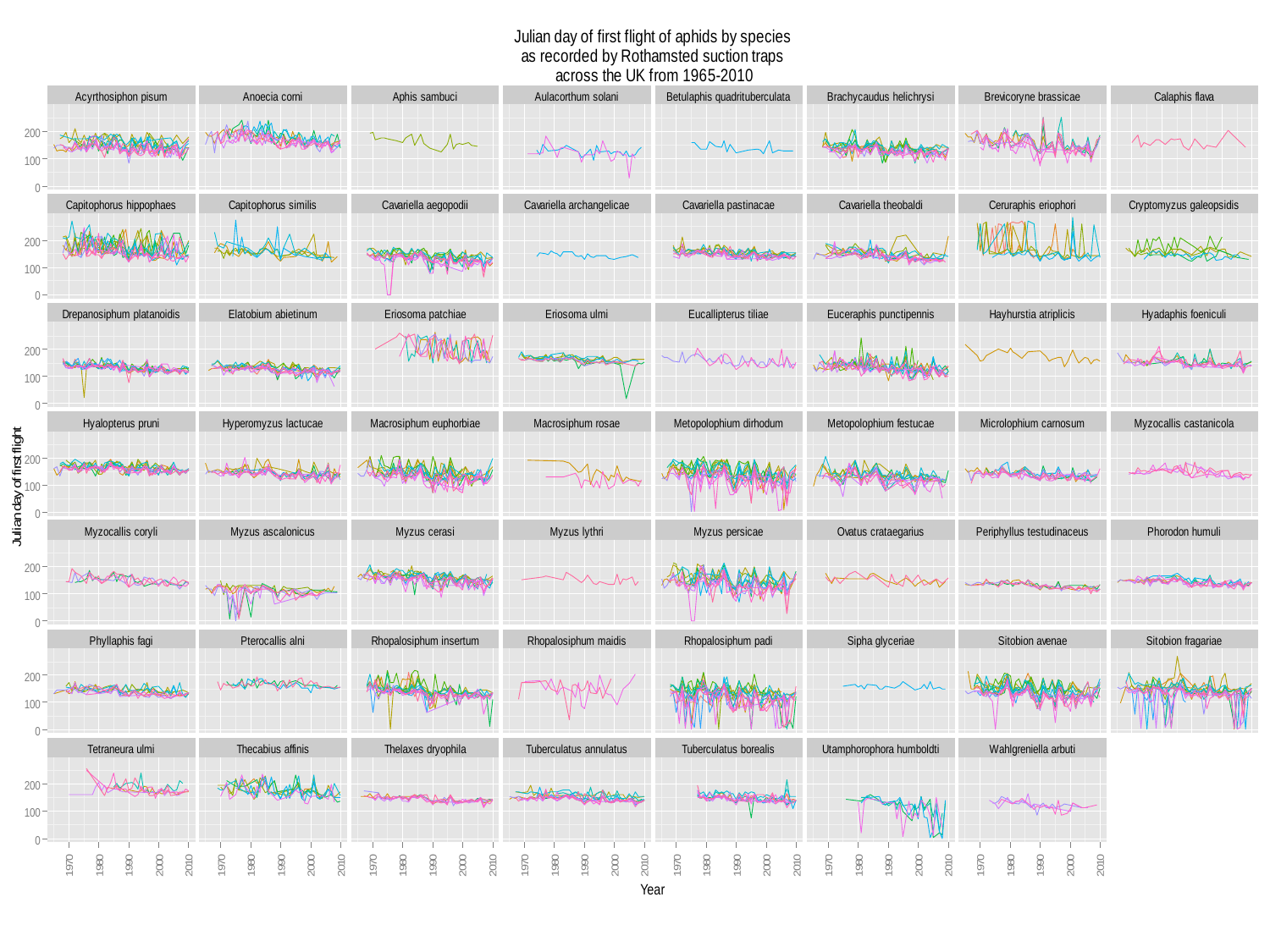

#

## Slide 3
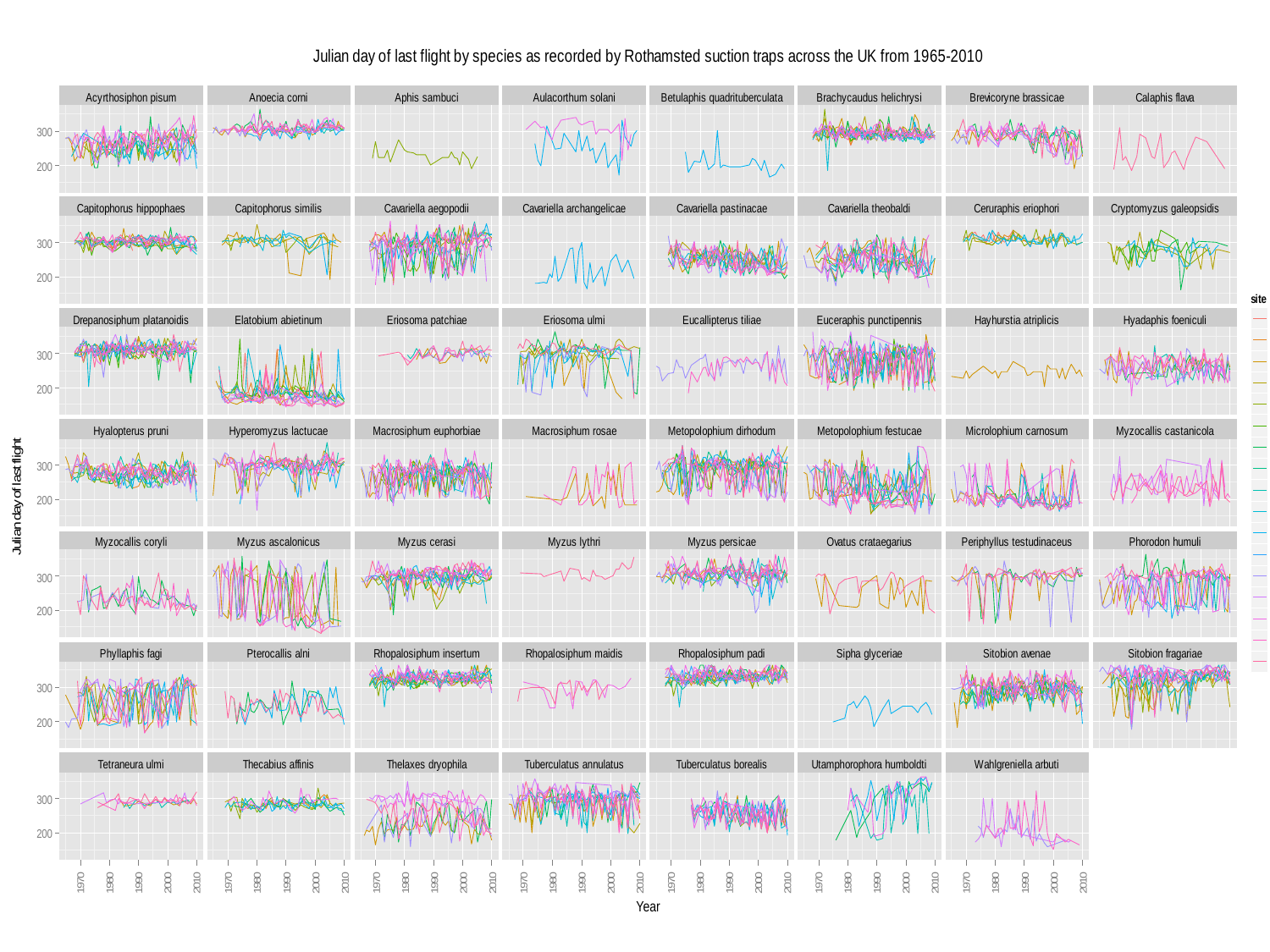

#

## Slide 4
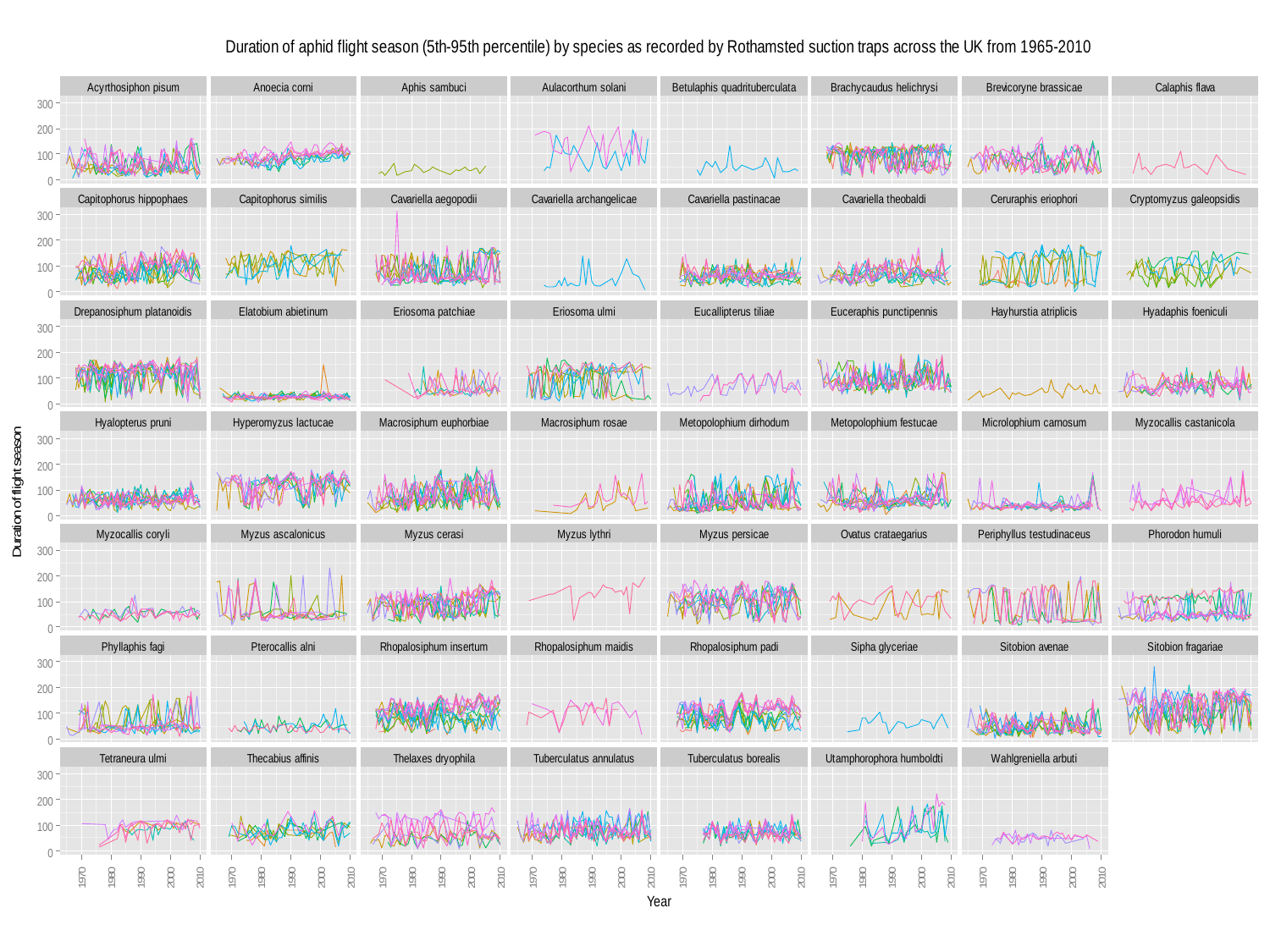

#

## Slide 5
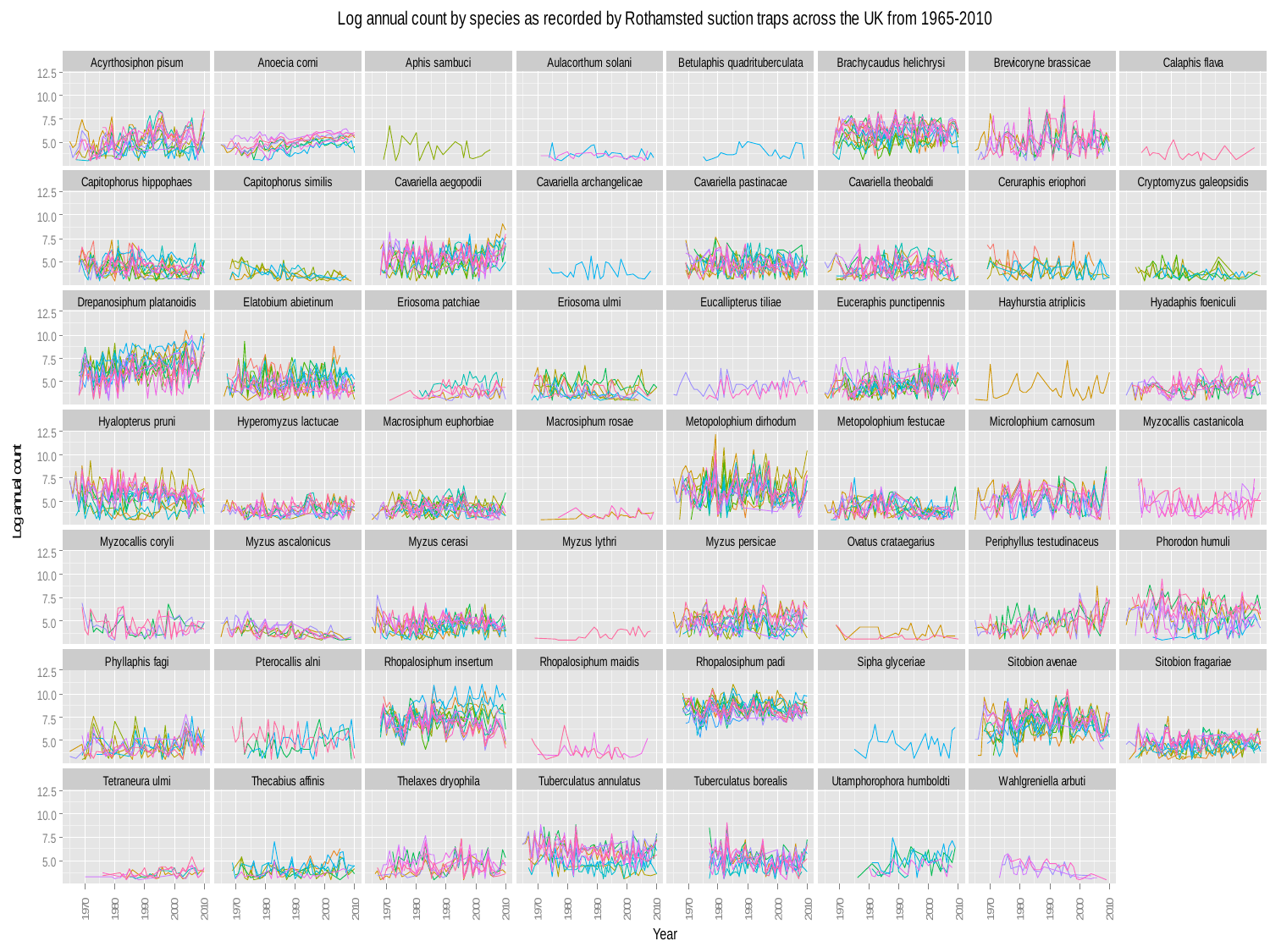

#

## Slide 6
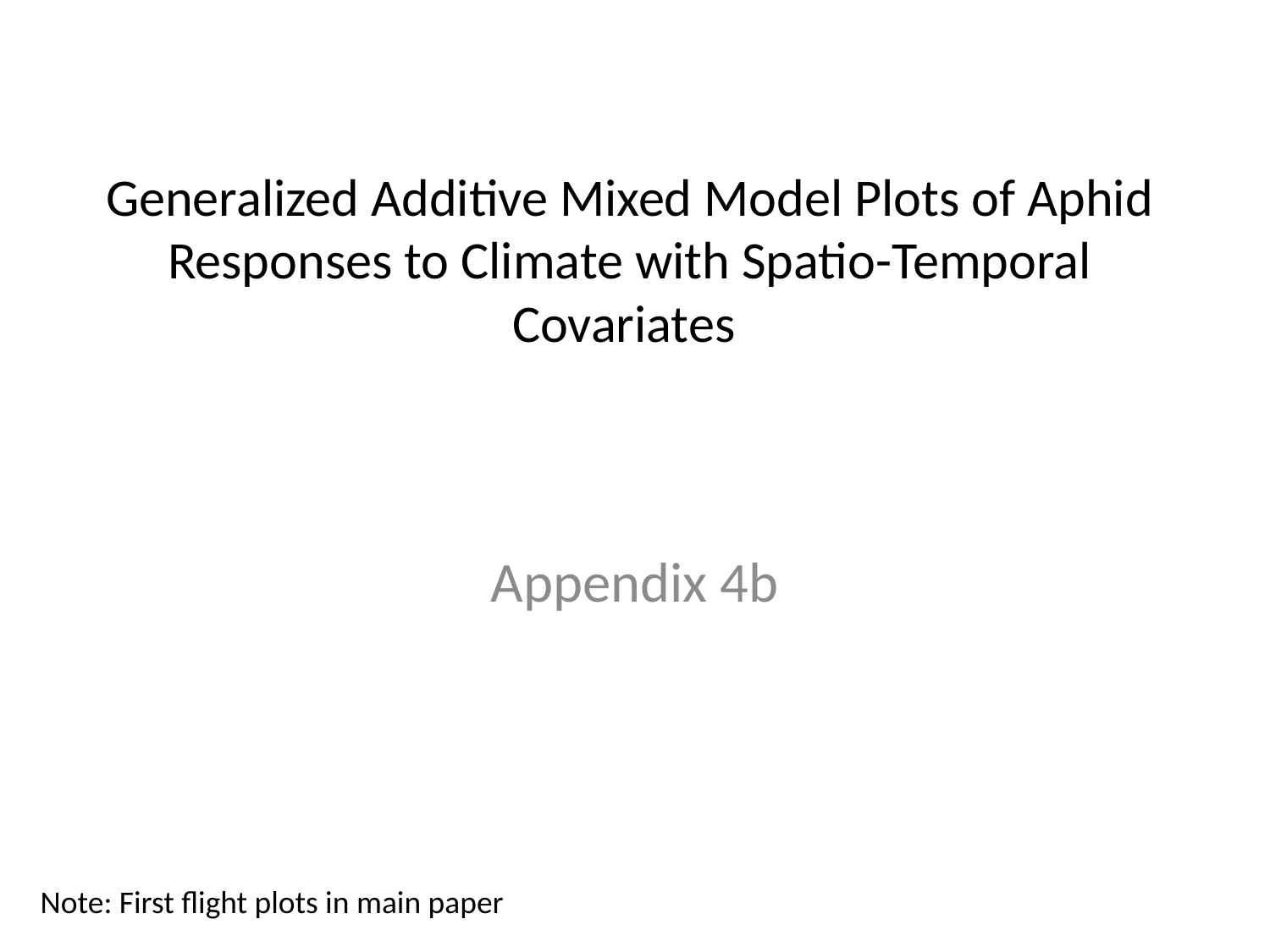

# Generalized Additive Mixed Model Plots of Aphid Responses to Climate with Spatio-Temporal Covariates
Appendix 4b
Note: First flight plots in main paper

## Slide 7
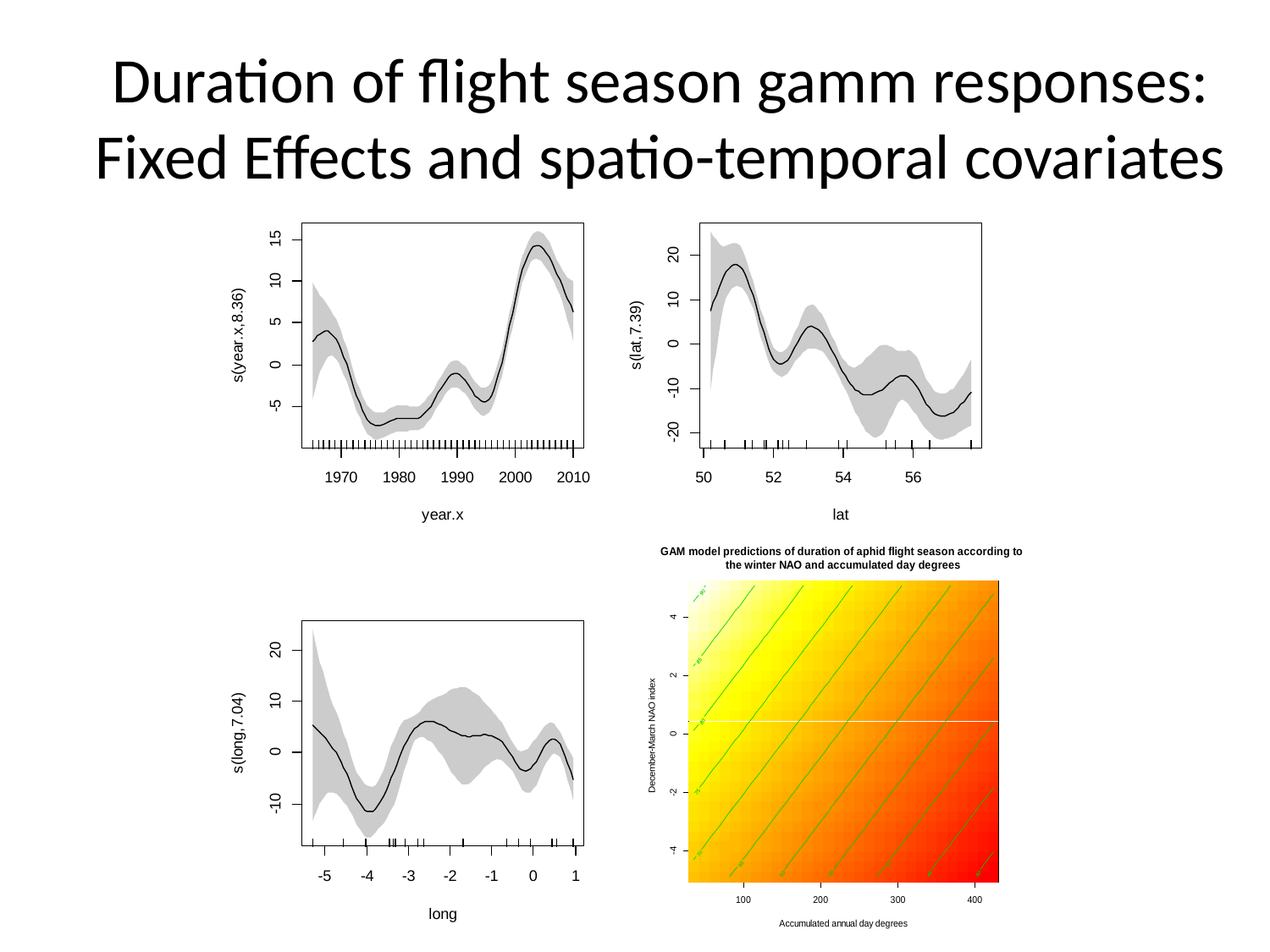

# Duration of flight season gamm responses: Fixed Effects and spatio-temporal covariates

## Slide 8
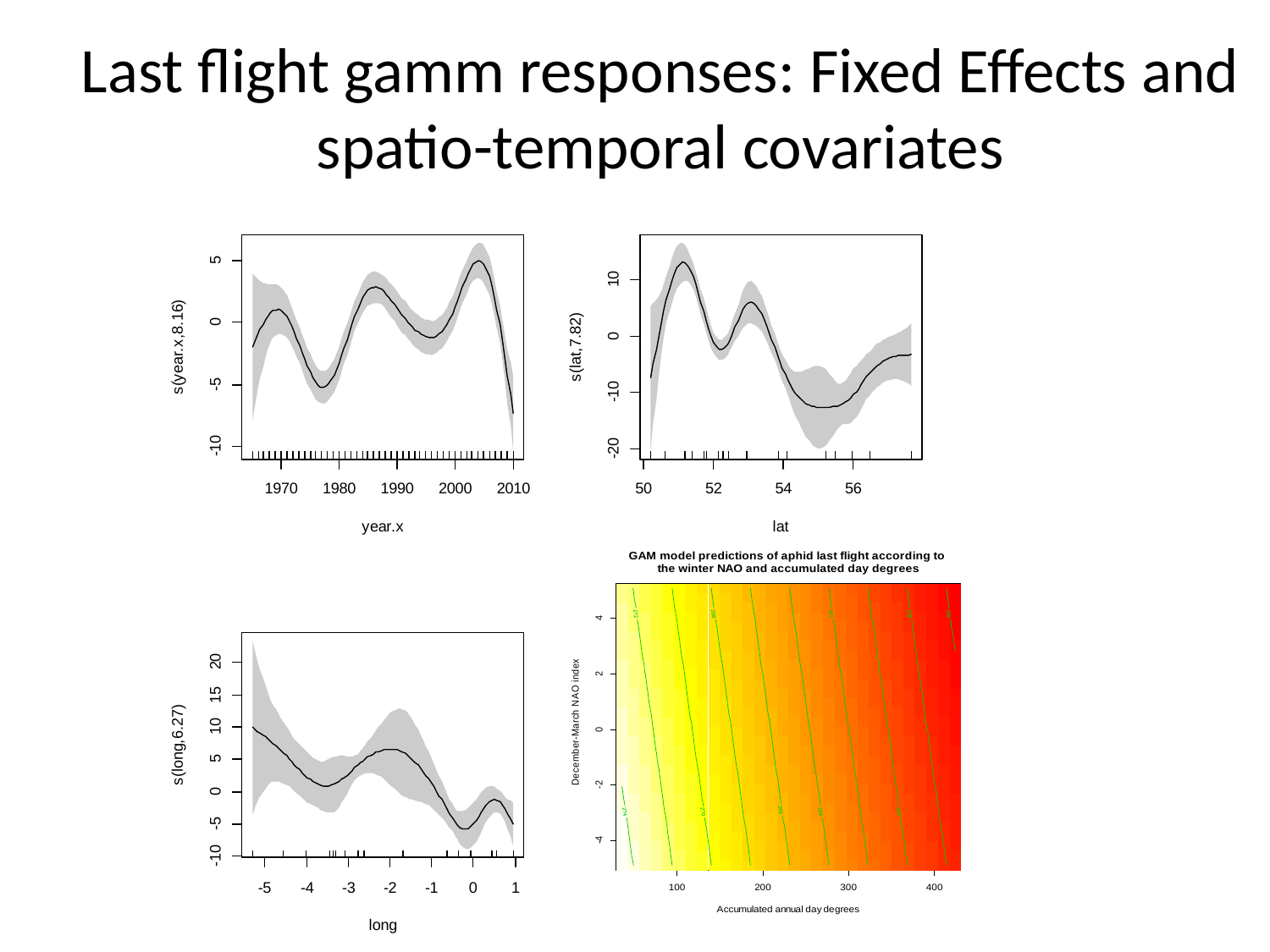

Last flight gamm responses: Fixed Effects and spatio-temporal covariates

## Slide 9
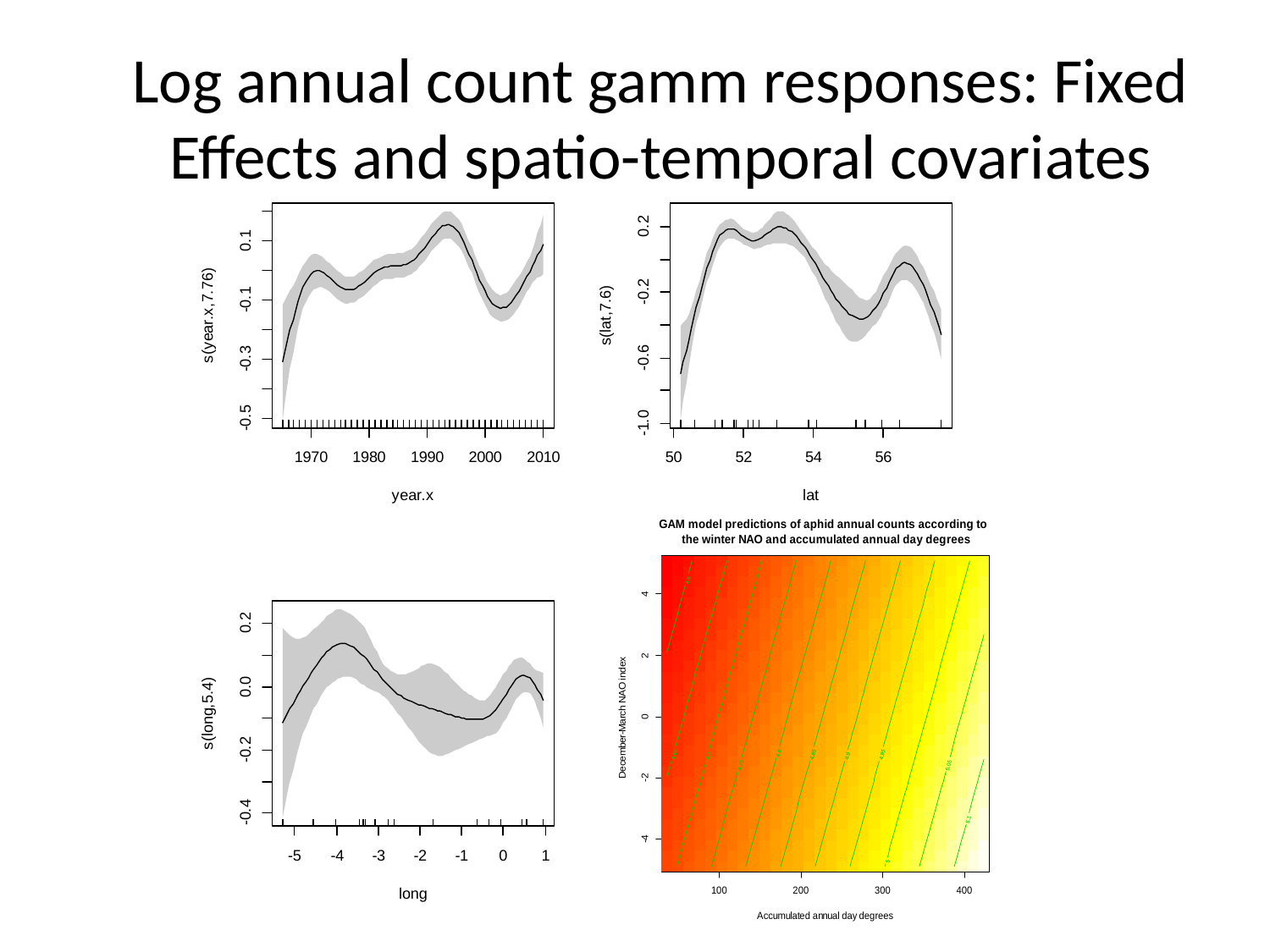

# Log annual count gamm responses: Fixed Effects and spatio-temporal covariates
