# Supplementary material for: Long-term phenological trends, species accumulation rates, aphid traits and climate: five decades of change in migrating aphids
Source: J Anim Ecol. 2014 Oct 3;84(1):21–34. doi: 10.1111/1365-2656.12282 (PMC4303923; doi:10.1111/1365-2656.12282)
Supplement: Supplementary file 5 — Appendix S5. Principal components analysis of linear mixed-effects model coefficients. [file jane0084-0021-sd5.pptx]

## Slide 1
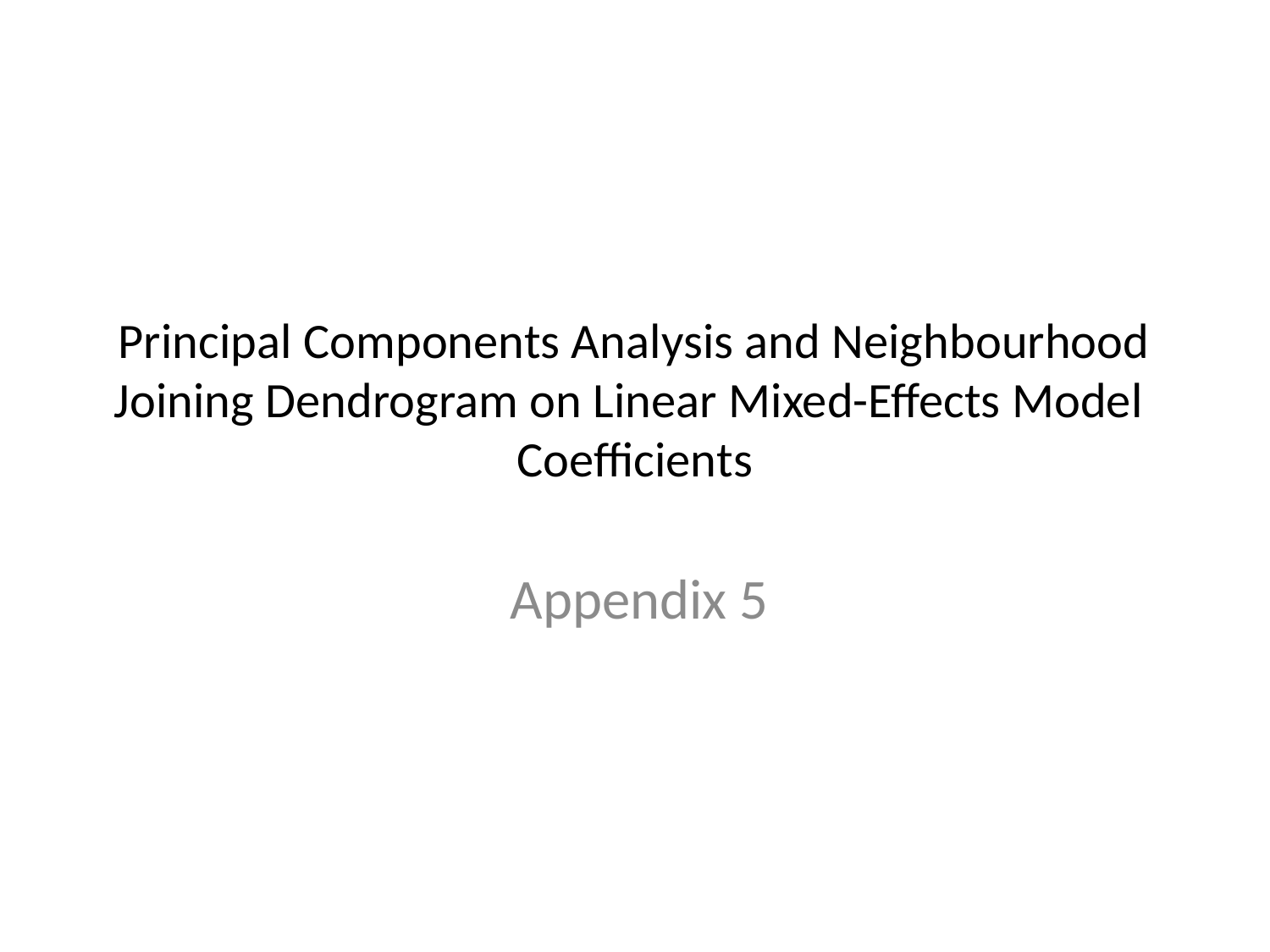

# Principal Components Analysis and Neighbourhood Joining Dendrogram on Linear Mixed-Effects Model Coefficients
Appendix 5

## Slide 2
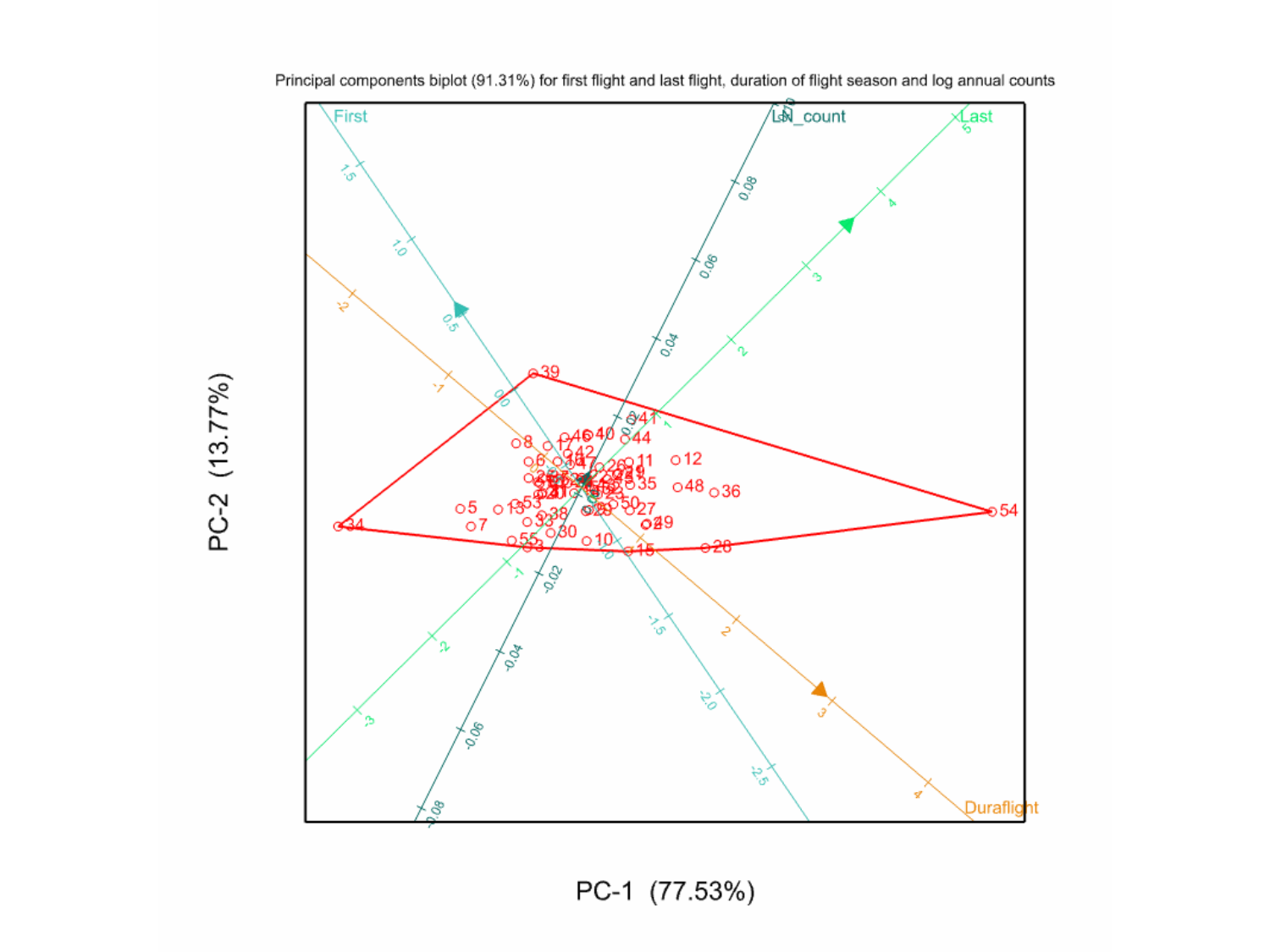

## Slide 3
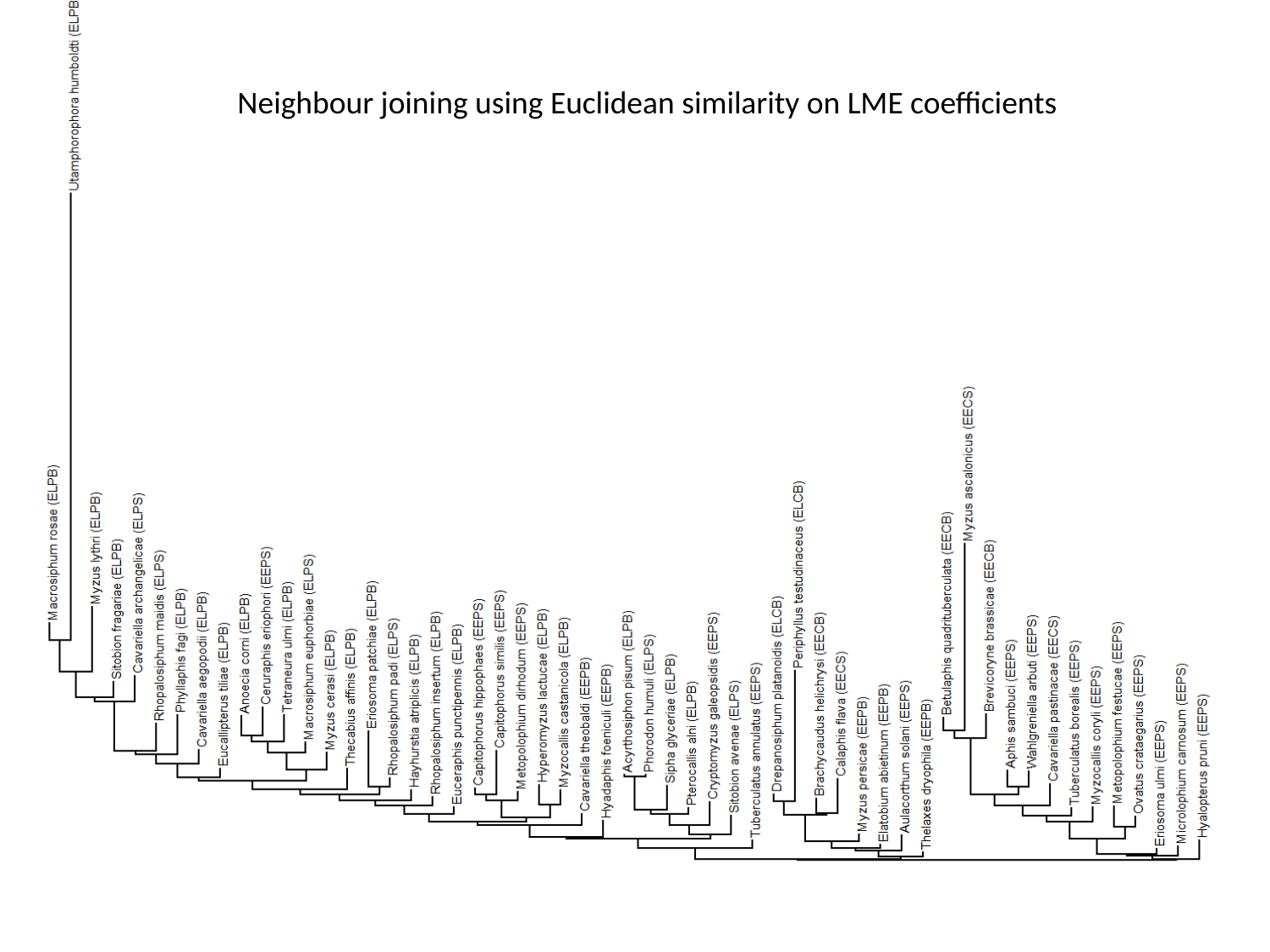

Neighbour joining using Euclidean similarity on LME coefficients
